# Geographic variation in evolutionary rescue under climate change in a crop pest–predator system

**DOI:** 10.1111/eva.13750

**Published:** 2024-07-22

**Authors:** Xuezhen Ge, Jonathan A. Newman, Cortland K. Griswold

**Affiliations:** ^1^ Department of Integrative Biology University of Guelph Guelph Ontario Canada; ^2^ Department of Biology Wilfrid Laurier University Waterloo Ontario Canada

**Keywords:** additive genetic variance, climate change, evolutionary rescue, predator–prey interaction, species distribution models, thermal performance

## Abstract

Species distribution models (SDMs) are often built upon the “niche conservatism” assumption, such that they ignore the possibility of “evolutionary rescue” and may underestimate species' future range limits under climate change. We select aphids and ladybirds as model species and develop an eco‐evolutionary model to explore evolutionary rescue in a predator–prey system under climate change. We model the adaptive change of species' thermal performances, accounting for biotic interactions. Our study suggests that, without considering evolutionary adaptation, the warming climate will result in a reduction in aphid populations and the extinction of ladybirds in large parts of the United States. However, when incorporating evolutionary adaptation into the model, aphids can adapt to climate change, whereas ladybirds demonstrate geographic variation in their evolutionary rescue potential. Specifically, ladybirds in southern regions are more likely to be rescued than those in the north. In certain northern regions, ladybirds do not avoid extinction due to severe warming trends and seasonality of the climate. While higher warming trends do prompt stronger evolutionary changes in phenotype, they also lead to reduced aphid population abundance such that ecology constrains ladybird population growth. Higher seasonality induces an ecological effect by limiting the length of reproductive season, thereby reducing the capacity for evolutionary rescue. Together, these findings reveal the complex interplay between ecological and evolutionary dynamics in the context of evolutionary adaptation to climate change.

## 1 INTRODUCTION

The response of species to climate change is a major concern in both applied and basic research. In the field of agriculture, a notable uncertainty is how insect pests will respond to a changing climate (Jactel et al., 2019; Skendžić et al., 2021). Insect pests, especially those with short generations, have the potential to undergo rapid evolutionary adaptation, making it challenging to estimate their potential distribution under climate change (Barzman et al., 2015; Lehmann et al., 2020; Sánchez‐Guillén et al., 2016). Although species distribution models (SDMs) have been widely applied by policy makers for pest management and risk assessment, evolution is typically overlooked in most of the previous studies (e.g., Bush et al., 2016; Smith et al., 2022). Furthermore, insect pests are inherently associated with their natural predators, forming predator–prey systems, and here again, SDMs are limited in their application (Kissling & Schleuning, 2015). When considering advancements in our understanding of species responses to climate change and SDM methodology, agricultural herbivore predator–prey systems form valuable model systems because often there are detailed experiments to support the development of mechanistic SDMs that account for evolutionary and ecological responses to climate change.

Species distribution models are widely used for exploring species' habitat suitability in response to climate change. Correlative SDMs commonly assume that ecological niches are conserved across space or over time (Soberón & Nakamura, 2009). “Niche conservatism” assumes that species retain their *fundamental* niche over time, and arises as an assumption in correlative SDMs that are based on observed present‐day associations between species presences and environments. This may be unrealistic because many species can rapidly evolve, especially those with short generations (Garnas, 2018). However, rapid evolution was widely ignored in application studies (Booth, 2017). Nevertheless, a variety of model frameworks have emerged to study species adaptation under climate change and incorporate eco‐evolutionary processes into SDMs, such as Allelic adaptive dynamics (ALADYN; Schiffers & Travis, 2014), RangeShifter (Bocedi et al., 2014), AdaptR (Bush et al., 2016), Gillespie eco‐evolutionary models (GEMs; DeLong & Gibert, 2016), Dynamic eco‐evolutionary models (DEEMs; Cotto et al., 2017), and a macroecological approach (Diniz‐Filho et al., 2019). In addition, a number of studies have utilized a ‘moving‐optimum’ model to investigate the evolution of quantitative traits (e.g., Kopp & Matuszewski, 2014). These models assume that the optimal values for a particular trait change over time, leading to changes including a shift in the mean phenotype and in phenotypic and possibly genotypic variance. Furthermore, integral projection models (Coulson et al., 2021) are able to model evolution under general conditions, such as non‐Gaussian phenotype distribution, as well as stage and age structure. Altogether, fundamental theoretical elements exist to develop detailed mechanistic models of species distributions. Here, we seek to advance the field of inquiry further by demonstrating how thermal performance evolution and predator–prey interactions affect species distributions.

**FIGURE 1:**
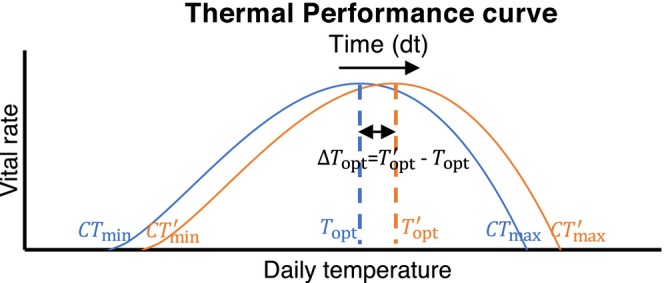
An illustration of trait evolution under climate change and its effect on species thermal performance. The blue and orange curves represent the thermal performance curves for species' vital rates. The optimum temperature evolves from $T_{\mathrm{opt}}$ to $T_{\mathrm{opt}}^{\prime }$ with the evolutionary adaptation in response to climate change.

Ectotherms, such as aphids and ladybirds experience changes in their vital rates as the environment moves between optimal and extreme conditions. These temperature‐dependent traits can be represented using Thermal Performance Curves (TPCs), which are captured in part by thermal optima $\left(T_{\mathrm{opt}}\right)$ and thermal tolerances or limits (${\mathrm{CT}}_{\min}$ and ${\mathrm{CT}}_{\max}$) (Sinclair et al., 2016). A species' TPC may evolve over time, impacting the fitness of the species (Figure 1). The suitability of a habitat for a species changes with its evolutionary response. The integration of TPC evolution is essential to understand ectotherm responses to climate change (e.g., Ge et al., 2023; Liao et al., 2021; Sinclair et al., 2016).

Aphids are sap‐sucking insects with direct negative effects on crop yields through feeding, and indirectly by being a vector of plant viruses (Koch et al., 2016). One major predator of aphids is the ladybird beetle, forming a natural top‐down regulation of aphid abundance. The life histories of aphids and ladybirds are quite different, for example, a shorter generation time for aphids versus ladybirds, and the aphid's use of both sexual and asexual reproduction (Morgan et al., 2001; Raak‐van den Berg et al., 2017). Aphids alternate between a long asexual phase and a short sexual phase (Simon et al., 2010). Without sexual reproduction, independent assortment and recombination, additive genetic variance may not be restored across a generation, affecting evolutionary response. In contrast, ladybirds reproduce continuously, although with a longer generation time compared to aphids. Together, the aphid‐ladybird system highlights key life‐history differences that may affect their respective evolutionary response.

We constructed a set of differential equations that are coupled with elements accounting for the evolutionary response in the optimal temperature ($T_{\mathrm{opt}}$) for both predator and prey. Unlike previous eco‐evolutionary approaches such as Abrams and Matsuda (1997), we allow for additive genetic variances of aphids and ladybirds to evolve with climate change using the infinitesimal approach of Bulmer (1971). For both aphids and ladybirds, combined population abundance and evolutionary response make use of an integral approach (motivated by Lande, 1979; Slatkin, 1970), whereby population abundance and evolutionary responses are a function of the distribution of breeding values for thermal performance traits and environmental effects. In the case of aphids, its response is not only a function of its distribution of breeding values and environmental effects, but also those of its predator abundance. We selected nine representative geographic locations in the United States at different latitudes and longitudes, and simulated the dynamics for 150 years, starting with the locally adapted optimal temperatures for the two species (aphid: $T_{\mathrm{opt},A}^{*}$; ladybird: $T_{\mathrm{opt},L}^{*}$). As part of our analysis, we explored two submodels, one without climatic change and evolution, and another with climate change but not evolution. By comparing the long‐term population dynamics of the two submodels with a full model that includes both climate change and evolution, we can evaluate the impact of climate change and evolution on predator and prey populations. Since our theoretical findings indicate that predators cannot be rescued in every geographic region, we further explored the mechanism of evolutionary rescue for the predator by comparing the roles of seasonality and warming trend on ecological outcomes. Together, our paper supports the advancement of SDMs to a broader set of conditions and systems.

## 2 METHODS

### 2.1 Biology of the aphid and ladybird

The life cycle of aphids includes both asexual and sexual generations. Aphids continually reproduce asexually during most of the year, and reproduce sexually once during the autumn (Dixon, 1977). For many aphid species, the occurrence of a sexual generation is triggered by environmental cues such as short‐day length and low temperatures (Ogawa & Miura, 2014). Ladybirds, on the other hand, only reproduce sexually and have a longer generation time than aphids (Pervez & Omkar, 2006). Ladybird predation of aphids follows a type II functional response (Sharma et al., 2017).

**FIGURE 2:**
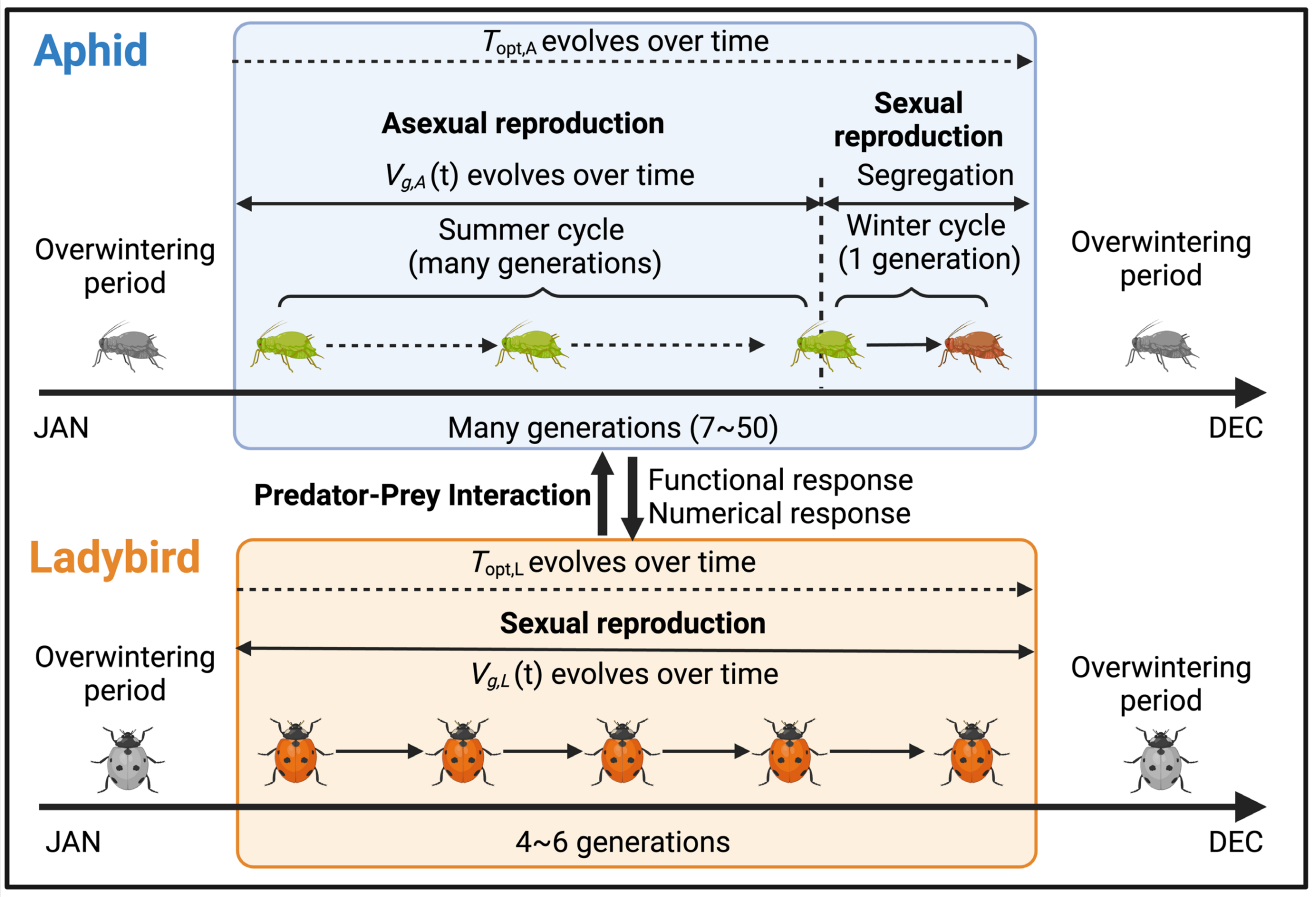
Schematic diagram of the life cycles for aphids and ladybirds within a year. Aphids continually reproduce asexually during most of the year, and reproduce sexually once in the autumn. Ladybirds reproduce sexually continuously. $T_{\mathrm{opt},A}$ and $T_{\mathrm{opt},L}$ evolve over time with evolutionary adaptation.

The vital rates for both aphids and ladybirds are sensitive to environmental temperatures (Hulle et al., 2010; Raak‐van den Berg et al., 2017). The shape parameters for the TPCs of these vital rates vary depending on the species and their developmental stages (Jalali et al., 2014; Zhao et al., 2017). Experimental studies on the thermal performance of aphids and ladybirds provide empirical support to parameterize our model (e.g., Morgan et al., 2001; Raak‐van den Berg et al., 2017). For simplicity, we assume the TPCs for aphids and ladybirds have the same functional shape. We assume these TPCs may shift horizontally with climate change, meaning that the three TPC parameters ${\mathrm{CT}}_{\min}$, ${\mathrm{CT}}_{\max}$ and $T_{\mathrm{opt}}$ evolve together at the same rate and direction (Figure 1). Thus, we substitute ${\mathrm{CT}}_{\min}$ and ${\mathrm{CT}}_{\max}$ with functions of $T_{\mathrm{opt}}$ to keep the evolutionary component concise. We applied non‐stage structured models for both aphids and ladybirds. We used the functions from Ge et al. (2023) (see Supplement S1, Equations S1 and S2), and estimated the shape parameters (see Table S1) by fitting the functions to life‐history data from aphids and ladybirds. Figure 2 illustrates the reproduction phases and their corresponding evolving traits.

### 2.2 Climate data for selected geographic locations

To examine the geographic variation in evolutionary response, we selected nine locations from agricultural regions in the United States (Table 1). These locations are evenly distributed across the area from 32° N to 44° N, and 84° W to 104° W, east of the Rocky Mountains. The location IDs in Table 1 indicate the relative positions of these places and serve as abbreviations for the locations in the subsequent content.

**TABLE 1:** Locations and temperature parameters of the nine geographical locations in the United States.

ID	Latitude	Longitude	Location	Tmean ($\bar{T}$)	Seasonality (s)	Trend (k)	${\bar{z}}_{A}^{*}$	${\bar{z}}_{L}^{*}$
				°C/year	°C/year	°C/100 years	°C	°C
SW	32° N	104° W	Angeles, Texas	13.318	12.398	7.814	22.5	22.0
SO	32° N	94° W	Logansport, Louisiana	16.889	10.904	5.779	24.5	24.5
SE	32° N	84° W	Cobb, Georgia	15.973	9.942	5.793	22.5	22.5
CW	38° N	104° W	Fowler, Colorado	5.055	13.699	8.109	16.0	15.5
CO	38° N	94° W	Taberville, Missouri	11.252	15.805	7.138	24.0	23.5
CE	38° N	84° W	Mt. Sterling, Kentucky	10.723	13.264	6.476	21.0	20.5
NW	44° N	104° W	West Pennington, South Dakota	4.153	16.159	7.734	17.0	16.5
NO	44° N	94° W	Beauford, Minnesota	5.103	19.705	8.290	19.0	18.5
NE	44° N	84° W	Standish, Michigan	6.477	15.917	7.579	19.0	19.0

*Note*: Seasonality is half of the difference between the yearly temperature minimum and maximum at a given location. The trend is the change in mean temperature over 100 years. These projections come from the French CNRM‐CM6‐1 model using the highest emission scenario (i.e., SSP5‐8.5; Coupled Model Intercomparison Project Phase 6 [CMIP6] Voldoire, 2018). For more information see http://www.umr‐cnrm.fr/cmip6/spip.php?article11. ${\bar{z}}_{A}^{*}$ and ${\bar{z}}_{L}^{*}$ represent the locally adapted $T_{\mathrm{opt}}$ for aphids and ladybirds in 2000. They are estimated by the procedures in Supplement S3.

To predict future climate, we used daily temperature data from a French General Circulation Model (CNRM‐CM6‐1) under the highest emission scenario (SSP5‐8.5) (Voldoire, 2018). Daily temperature time series data were decomposed into trend, seasonality, and noise by spectral analysis, with noise subsequently removed. The two remaining components constitute our “smoothed” temperature data, capturing the seasonality and the warming trend of the local climate. We used smoothed temperature to eliminate the effect of day‐to‐day variability; implying an inherent physiological buffering against day‐to‐day variability.

To simulate the daily mean temperature for each geographic location, we adopted three climate parameters, which are $\bar{T}$, s and k. In Equation (1), (2), (3), t denotes time (day), t=1 represents Jan. 1st, 2000 (day 1). $\bar{T}$ denotes the annual mean temperature in 2000 (Equation 1). k denotes the trend of temperature change over the century, kt/36500 represents the accumulated temperature change over time (each year is assumed to have 365 days, with no consideration for leap years). $\bar{T}$ and k were fit by linear regression using the “trend” component of the decomposed time series temperature data. s denotes the ‘seasonality’ of daily temperature, defined as half of the difference between yearly minimum ($T_{\min,\text{year}}$) and maximum temperatures ($T_{\max,\text{year}}$; Equation 2). It was calculated by estimating the amplitude of the seasonality component. Then, we used a cosine function (Equation 3, similar to Equation S1 in Ge et al., 2023) to generate the daily temperature time series for 2000–2150 at each location. The temperature parameter values of the nine locations are listed in Table 1.

(1) $$\bar{T}=\frac{\int_{1}^{365}T_{t}\;dt}{365},$$

(2) $$s=\left(T_{\max,\text{year}}-T_{\min,\text{year}}\right)/2,$$

(3) $$T_{t}=-s\cos\left(\frac{2\mathrm{\pi t}}{365}\right)+\bar{T}+\mathrm{kt}/36500.$$

### 2.3 Model description

#### 2.3.1 Population dynamics and evolution

For the *i*th individual aphid and the *j*th ladybird, phenotypic values ($z_{A,i}$, $z_{L,j}$) of the optimum temperature ($T_{\mathrm{opt}}$) are determined by the corresponding additive genetic effects ($z_{g,A,i}$, $z_{g,L,j}$) and the environmental effects ($z_{e,A,i}$, $z_{e,L,j}$),

(4) $$z_{A,i}=z_{g,A,i}+z_{e,A,i},$$

(5) $$z_{L,j}=z_{g,L,j}+z_{e,L,j}.$$

Vital rates of each individual are functions of air temperature $\left(T\right)$ and phenotypic value $\left(z_{A,i},,,\;z_{L,i}\right)$. Population sizes for ladybirds $\left(L\right)$ and aphids $\left(A\right)$ are modeled according to the following system of ordinary differential equations:

(6) $$\frac{dA}{dt}=\bar{f}\left(\left\{z_{A,i}\right\},T,t\right)\left(1-\frac{A}{K}\right)A-\bar{\beta }\left(\left\{z_{L,j}\right\},T,t\right)\mathrm{AL}-\bar{\mu }\left(\left\{z_{A,i}\right\},T,t\right)A,$$

(7) $$\frac{dL}{dt}=\frac{\bar{\beta }\left(\left\{z_{L,j}\right\},T,t\right)\mathrm{AL}}{Q_{p}}-\bar{\gamma }\left(\left\{z_{L,j}\right\},T,t\right)L,$$

where $\bar{f}\left(\left\{z_{A,i}\right\},T,t\right)$ represents the mean temperature‐dependent birth rate for the aphid population at time t, and $\bar{\mu }\left(\left\{z_{A,i}\right\},T,t\right)$ and $\bar{\gamma }\left(\left\{z_{L,j}\right\},T,t\right)$ represent the mean intrinsic mortality rates for aphids and ladybirds at time t, respectively. Within each of these functions, $\left\{z_{A,i}\right\}$ and $\left\{z_{L,j}\right\}$ indicate the continuous set of phenotypes in the population for aphids and ladybirds, respectively. K is the carrying capacity for the aphid population. For the prey, we assume that ladybird predation is the only source of extrinsic mortality, and intrinsic mortality is a function of phenotype and temperature. For the predator, we assume that fecundity rates depend on prey capture rates, and intrinsic mortality is a function of phenotype and temperature. Predator–prey interactions are determined by the functional and numerical responses of ladybirds. Previous research has suggested that a Type II functional response is commonly observed in ladybirds (e.g. Sharma et al., 2017). We modified Holling's (1959) Type II functional response, by adding the function $g\left(z_{L,j},T,t\right)$ to model the effect of temperature:

(8) $$\beta \left(z_{L,j},T,t\right)=g\left(z_{L,j},T,t\right)\frac{a}{\left(1+\mathrm{ahA}\right)},$$

where a and h denote predator's searching rate and handling time, respectively. $g\left(z_{L,j},T,t\right)$ (see Supplement S1) exhibits a unimodal asymmetric shape on the unit interval. $\beta \left(z_{L,j},T,t\right)$ represents the rate each aphid is predated by each ladybird, which is a function of aphid population density and temperature. Thus, in Equation 7, $\bar{\beta }\left(\left\{z_{L,j}\right\},T,t\right)$ represents the mean predation effect on aphid population at time t. We assume that the predator's numerical response is proportional to the number of prey consumed (Solomon, 1949). The transformation rate ($Q_{p}$ in Equation 7) represents the mean number of aphids a ladybird needs to consume to reproduce a single egg. Thus, the number of consumed prey contributing to population growth of predators is $\frac{\bar{\beta }\left(\left\{z_{L,j}\right\},T,t\right)\mathrm{AL}}{Q_{p}}$ (Equation 7). For parameter values see Supplement S1 and Table S1.

From Equation 6, for an individual aphid with phenotype $z_{A,i}$ and interacting with a ladybird with phenotype $z_{L,j}$, the instantaneous rate of growth of its descendant lineage at time t is

(9) $$w_{A}\left(z_{A,i},z_{L,j},t\right)=\underset{F\left(z_{A,i},t\right)}{\underbrace{f\left(z_{A,i},T,t\right)\left(1-\frac{A}{K}\right)-\mu \left(z_{A,i},T,t\right)}}-\underset{B\left(z_{L,j},t\right)}{\underbrace{g\left(z_{L,j},T,t\right)\frac{\mathrm{aL}}{\left(1+\mathrm{ahA}\right)}}}$$

(10) $$=F\left(z_{A,i},t\right)-B\left(z_{L,j},t\right).$$

Note that $F\left(z_{A,i},t\right)$ or $B\left(z_{L,j},t\right)$ are functions of A, L, and T, but for brevity, they are suppressed in the following notations.

The instantaneous rate of growth of the descendant lineage for a ladybird with phenotype $z_{L,j}$ is

(11) $$w_{L}\left(z_{L,j},t\right)=g\left(z_{L,j},T,t\right)\frac{\mathrm{aA}}{\left(1+\mathrm{ahA}\right)Q_{p}}-\gamma \left(z_{L,j},T,t\right).$$

Recall for a species that does not vary in phenotype, its population density $N\left(t+\Delta t\right)$ after a time interval Δt is $N\left(t\right)+\mathrm{rN}\left(t\right)\Delta t$ (r is the instantaneous rate of growth). In an analogous manner, and focusing on the aphid species at time t+Δt, an individual with phenotype $z_{A,i}$ is expected to be at density:

(12) $$A\left(z_{A,i},t+\Delta t\right)=\rho \left(z_{A,i},t\right)A\left(t\right)+\rho \left(z_{A,i},t\right)\int w_{A}\left(z_{A,i},,,z_{L},,,t\right)\rho \left(z_{L},,,t\right)\;dz_{L}A\left(t\right)\Delta t,$$

where $\rho \left(z_{A,i},t\right)$ is the probability density of an aphid individual with phenotype $z_{A,i}$ at time t, and $\rho \left(z_{L},,,t\right)$ is the corresponding probability density for ladybirds. Across all aphid and ladybird individuals with different phenotypes ($z_{A,i}$ and $z_{L,j}$), the expected size of the aphid population is

(13) $$A\left(t+\Delta t\right)=A\left(t\right)+\iint w_{A}\left(z_{A},z_{L},t\right)\rho \left(z_{A},,,t\right)\rho \left(z_{L},,,t\right)A\left(t\right)\Delta t\;dz_{A}dz_{L}$$

(14) $$=A\left(t\right)+A\left(t\right)\Delta t\left(\underset{\text{Denoted}\;\mathrm{as}\;{\bar{F}}_{A}\left(t\right)}{\underbrace{\int F\left(z_{A},t\right)\rho \left(z_{A},t\right)\;dz_{A}}}-\underset{\text{Denoted}\;\mathrm{as}\;{\bar{B}}_{L}\left(t\right)}{\underbrace{\int B\left(z_{L},t\right)\rho \left(z_{L},t\right)\;dz_{L}}}\right)$$

(15) $$=A\left(t\right)+A\left(t\right)\Delta t\left({\bar{F}}_{A}\left(t\right)-{\bar{B}}_{L}\left(t\right)\right)$$

(16) $$=A\left(t\right)\left(1+\left({\bar{F}}_{A}\left(t\right)-{\bar{B}}_{L}\left(t\right)\right)\Delta t\right).$$

The expected size of the ladybird population is

(17) $$L\left(t+\Delta t\right)=L\left(t\right)\left(1+{\bar{w}}_{L}\left(t\right)\Delta t\right),$$

where ${\bar{w}}_{L}$ is defined as $\int w_{L}\left(z_{L},,,t\right)\rho \left(z_{L},,,t\right)\;dz_{L}$. $z_{A}$ and $z_{L}$ represent phenotypic values, which are sums of additive genetic and environmental effects, such that integrations are over the distributions of $z_{g,s}$ and $z_{e,s}$ with means ${\bar{z}}_{g,s}$ and ${\bar{z}}_{e,s}=0$, and variances $V_{g,s}$ and $V_{e,s}$, where ${\bar{z}}_{g,s}$ and ${\bar{z}}_{e,s}$ represent the mean breeding and environmental values for aphids (s=A) and ladybirds (s=L) at time t, $V_{g,s}$ and $V_{e,s}$ represent the additive genetic and environmental variances for aphids and ladybirds, respectively. (Note that we use $V_{g}$ instead of $V_{A}$ in this study to represent additive genetic variance because we use ‘A' to represent aphid.) Across additive genetic and environmental components,

(18) $${\bar{F}}_{A}\left(t\right)=\iint F\left(z_{g,A}+z_{e,A},t\right)\rho \left(z_{g,A},t\right)\rho \left(z_{e,A},t\right)\;dz_{g,A}dz_{e,A},$$

(19) $${\bar{B}}_{L}\left(t\right)=\iint \beta \left(z_{g,L}+z_{e,L},t\right)\rho \left(z_{g,L},t\right)\rho \left(z_{e,L},t\right)\;dz_{g,L}dz_{e,L},$$

(20) $${\bar{w}}_{L}\left(t\right)=\iint w_{L}\left(z_{g,L}+z_{e,L},t\right)\rho \left(z_{g,L},t\right)\rho \left(z_{e,L},t\right)\;dz_{g,L}dz_{e,L},$$

where $\rho \left(z_{g,s},t\right)$ and $\rho \left(z_{e,s},t\right)$ represent the probability densities of aphid/ladybird individuals with additive genetic effect $z_{g,s}$ and environmental effect $z_{e,s}$ at time t, respectively. As we assume environmental effects are normally distributed, the probability density for each variable follows its corresponding normal distribution $N\left(z_{e},;,{\bar{z}}_{e},;,V_{e}\right)$, which denotes a random variable $z_{e}$ with the mean ${\bar{z}}_{e}$ and variance $V_{e}$.

Motivated by the integral approaches of Slatkin (1970) and Lande (1979), the distributions of breeding values for aphids and ladybirds after selection are

(21) $$\rho \left(z_{g,A},t+\Delta t\right)=\frac{\rho \left(z_{g,A},t\right)\left(1+\Delta t\int F\left(z_{g,A,t}+z_{e,A,t},t\right)\rho \left(z_{e,A},t\right)\;dz_{e,A}-\Delta t{\bar{B}}_{L}\left(t\right)\right)}{1+\left({\bar{F}}_{A}\left(t\right)-{\bar{B}}_{L}\left(t\right)\right)\Delta t},$$

(22) $$\rho \left(z_{g,L},t+\Delta t\right)=\frac{\rho \left(z_{g,L},t\right)\left(1+\Delta t\int w_{L}\left(z_{g,L}+z_{e,L},t\right)\rho \left(z_{e,L},t\right)\;dz_{e,L}\right)}{1+{\bar{w}}_{L}\left(t\right)\Delta t}.$$

For derivation see Supplement S2.

##### Evolving $V_{g}$ and $z_{g}$ for ladybirds

After selection (over time interval Δt), the mean breeding value (${\bar{z}}_{g,L}^{\prime }$) and additive genetic variance for ladybirds are

(23) $${\bar{z}}_{g,L}^{\prime }=\int z_{g,L}\rho \left(z_{g,L},t+\Delta t\right)\;{\mathrm{dz}}_{g,L},$$

(24) $$V_{g,L}^{\prime }=\int {\left(z_{g,L}-{\bar{z}}_{g,L}^{\prime }\right)}^{2}\rho \left(z_{g,L},t+\Delta t\right)\;{\mathrm{dz}}_{g,L}$$

(25) $$V_{G,L}^{\prime }=V_{g,L}^{\prime }+V_{\tau ,L}\Delta t,$$

where $V_{G,L}^{\prime }$ represents additive genetic variance plus mutational variance. Mutational variance is proportional to $V_{e,s}$, such that $V_{m,s}$ = $h_{m,s}^{2}$ $V_{e,s}$ (Lynch & Walsh, 1998, p. 338), where $h_{m,s}^{2}$ is the mutational heritability. Mutational variance is measured on a per generation timescale (Lynch & Walsh, 1998, p. 329). To transform mutational variance to match the timescale of this model, we multiplied $V_{m,s}$ by a coefficient $\eta_{s}$ with units of generations per time interval, such that $V_{\tau ,s}=\eta_{s}V_{m,s}$. The values $h_{m,s}^{2}$ and $\eta_{s}$ for both species are listed in Table S1.

The change in genetic variance over time interval Δt is

(26) $$\Delta V_{g,L}=V_{G,L}^{\prime }-V_{g,L}.$$

Selection can cause a change in genetic variance $\left(\Delta V_{g,L}\right)$ over time interval Δt, but continuous sexual reproduction can restore this change due to segregation (Bulmer, 1971). Thus, the mean breeding value and additive genetic variance for ladybirds after selection and random mating at time t+Δt is

(27) $${\bar{z}}_{g,L,t+\Delta t}={\bar{z}}_{g,L}^{\prime },$$

(28) $$V_{g,L,t+\Delta t}=V_{g,L,0}+d_{L,t+\Delta t},$$

(29) $$d_{L,t+\Delta t}=\frac{1}{2}d_{L,t}+\frac{1}{2}h_{t}^{4}\mathrm{dV}.$$

In Equation 28, $V_{g,L,0}$ is the segregation variance of ladybirds and is assumed to be constant under the infinitesimal genetic model. We assume ladybirds do not experience inbreeding. $d_{L,t+\Delta t}$ is the deviation from segregation variance due to selection $\left(d_{L,0}=0\right)$. As noted by Bulmer (1971, p. 203, Equation 8), dV is defined as the change in phenotypic variance and the term $\frac{1}{2}h_{t}^{4}\mathrm{dV}$ is equal to the change in genetic variance, such that we substitute $\Delta V_{g,L,t}$ (Equation 26) for $\frac{1}{2}h_{t}^{4}\mathrm{dV}$ in Equation 29. Under the infinitesimal genetic model, breeding values reset to be normally distributed with mean ${\bar{z}}_{g,L,t}$ and variance $V_{g,L,t}$. This distribution is used in Equations 19, 20 and 22 to obtain the probability density of ladybird individuals, $\rho \left(z_{g,L},t+\Delta t\right)$, using Equation 22.

##### Evolving $V_{g}$ and $z_{g}$ for aphids

Unlike ladybirds, aphids only reproduce sexually once before they overwinter each year. During the asexual phase, aphids undergo a form of stabilizing selection with a directional trend, which decreases $V_{g,A}$. Furthermore, because there is no segregation until the sexual phase, the distribution of breeding values may deviate from a normal distribution as selection accumulates. Accordingly, we track the distribution of breeding values at each time step and carry it through to the next, until the sexual phase (Equation 21). Although there is no new segregation variance generated during the asexual phase, there is the generation of variance by mutation. With the inclusion of mutational variance, the breeding values' distribution is adjusted to

(30) $$\rho \left(z_{G,A},t+\Delta t\right)=\int \rho \left(z_{g,A},t+\Delta t\right)\rho_{\tau }\left(z_{G,A}-z_{g,A}\right)\;{\mathrm{dz}}_{g,A},$$

where $z_{G,A}$ represents the genotypic value with mutational variance. $z_{G,A}-z_{g,A}$ denotes the mutational effect. $\rho_{\tau }$ is the probability density for the mutational effect, which follows a normal distribution $N\left(z_{G,A}-z_{g,A};0,V_{\tau }\right)$.

Thus, the mean breeding value $\left({\bar{z}}_{g,A}^{\prime }\right)$ and additive genetic variance $\left(V_{G,A}^{\prime }\right)$ for aphids after selection at time t+Δt (during the asexual phase) is

(31) $${\bar{z}}_{G,A}^{\prime }=\int z_{G,A}\rho \left(z_{G,A},t+\Delta t\right)\;{\mathrm{dz}}_{\mathrm{gG},A},$$

(32) $$V_{G,A}^{\prime }=\int {\left(z_{G,A}-{\bar{z}}_{G,A}^{\prime }\right)}^{2}\rho \left(z_{G,A},t+\Delta t\right)\;{\mathrm{dz}}_{G,A}.$$

Thus, the change in mean genetic variance over the asexual phase in year l is

(33) $$\Delta V_{g,A,l}=V_{G,A,l}^{\prime }-V_{G,A,l-1}^{\prime },$$

where $V_{G,A,l}^{\prime }$ and $V_{G,A,l‐1}^{\prime }$ represent the additive genetic variance of aphids at the end of the asexual phase in year l and l−1, respectively.

As aphids switch to the sexual phase, $V_{g,A}$ will be partially restored due to meiotic segregation, recombination, and random mating. Thus, the additive genetic variance after selection during the whole growing season and random mating in each year (l) is

(34) $$V_{g,A,l}=\left(1-f\right)V_{g,A,0}+d_{A,l}+V_{\tau ,A},$$

(35) $$d_{A,l}=\frac{\left(1-f\right)}{2}d_{A,l-1}+\frac{1}{2}h_{l-1}^{4}\mathrm{dV}.$$

Here, $V_{g,A,0}$ is the segregation variance of aphids and is constant under the infinitesimal genetic model. There is evidence that aphids undergo inbreeding during the sexual phase, which is captured by the term $\left(1-f\right)$, Barton et al. (2017), where f is the inbreeding coefficient. Empirical estimates of f are approximately 0.1 (Margaritopoulos et al., 2009; Wang et al., 2017), and this value was used in simulations. As before, $d_{A,l}$ is the deviation from segregation variance due to selection ($d_{A,0}=0$) (Bulmer, 1971), the term $\frac{1}{2}h_{l-1}^{4}\mathrm{dV}$ is equal to the change in genetic variance (Equation 33).

#### 2.3.2 Model sets

We construct three models to investigate the effects of climate change and evolutionary adaptation on the population dynamic of aphids and ladybirds. By decomposing the individual contribution of each factor, we are able to gain a better understanding of their effects on the predator–prey system and the complex interactions between these factors.

##### Model 1: No evolution and a stable climate

We simulate a stable climate where the daily temperatures have an annual pattern, but the pattern does not change through time (i.e., no interannual variation). Thus, the aphids and ladybirds do not experience the effects of climate change. We find values ${\bar{z}}_{A}^{*}$ and ${\bar{z}}_{L}^{*}$ (Table 1), such that they are at equilibrium for the local climates (See Supplement S3 for details). All of the rates described in Equations 6 and 7 are temperature‐dependent but have the same annual patterns each year. This provides a baseline of predator and prey dynamics in a constant environment, and can be used to compare the effects of climate change and evolution on these dynamics.

##### Model 2: No evolution, but with climate change

In this model, we assume aphids and ladybirds experience climate change, unlike the stable environment assumed in the baseline model. However, their thermal performances are not able to adapt evolutionarily to the changing conditions, i.e., $V_{g,A,0}=V_{g,L,0}=0$. As a result, the mean phenotypic values (${\bar{z}}_{A}$ and ${\bar{z}}_{L}$) remain constant after initially finding their optimal values for the year 2000. Despite this, the annual patterns of these rates will still be affected by the interannual variation in temperature data, which alters the annual population dynamics of aphids and ladybirds.

##### Model 3: Eco‐evolutionary model with climate change

In this model, we incorporate species' evolutionary adaption by allowing ${\bar{z}}_{A}$ and ${\bar{z}}_{L}$ to change through time in response to climatic change, i.e., $V_{g}>0$ for aphids and ladybirds. From Logan et al. (2020), we set $V_{g,L,0}=0.3$ and $V_{e,L}=0.7$ based on the estimated additive genetic variance and heritability for the thermal performance of the harlequin beetle (*Harmonia axyridis*). Equivalent data for aphids is lacking, so we assume that $V_{g,A,0}=V_{g,L,0}$ and $V_{e,A}=V_{e,L}$. The evolving mean phenotypic values and additive genetic variances for aphids and ladybirds are given by Equations (28), (29), (30), (31), (32), (33), (34).

### 2.4 Model simulations

#### 2.4.1 Long‐term evolutionary effects on population dynamics

For each location, we used temperature profiles for that location to run the three models described in Section 2.3.2 from 2000 to 2150. Initial conditions were the same for each model (A = 1,000,000, L = 5000, K = 50,000,000, ${\bar{z}}_{A}={\bar{z}}_{A}^{*}$, ${\bar{z}}_{L}=z_{L}^{*}$), and accounted for the overwintering periods for both species (Ge et al., 2023). At the beginning of each year, we introduce the aphids when the temperature is warm enough to support positive aphid growth rates ($w_{A}>1$). We then introduce ladybirds into the model when $w_{L}>1$. Daylength ($\mathrm{DL}\left(\phi \right)$) represents the number of daylight hours at different latitudes (ϕ) and is calculated according to the method described by Langille et al. (2016). During the growing season, aphids switch from asexual to sexual reproduction when the temperature (T, Equation 36) cools and daylength (DL, Equation 36) becomes shorter (Ogawa & Miura, 2014). Thus, we use ε to act as a computational control “switch” to enable the sexual phase. Aphids remain in their asexual phase when ε=0 and switch to their sexual phase when ε=1:

(36) $$\varepsilon =\left\{\begin{array}{cc} 1 & \text{if}\;\mathrm{DL}\left(\phi \right)<-0.05T+15.67\;\text{and}\;T<z_{A},\\ 0 & \text{otherwise}. \end{array}\right.$$

For simplicity, we use the date when aphids switch phases as the date when both species enter overwintering periods. During overwintering, aphids and ladybirds terminate reproduction and pass through the cold winter. Owing to the challenges in estimating species' overwinter survival, we assume an annual overwintering mortality rate of 0.2 for both species.

After both species enter the overwintering period: (*i*) both species stop their development, (*ii*) the mean phenotypes for aphid and ladybird remain unchanged until they emerge in the next year, and (*iii*) the additive genetic variance for aphids $\left(V_{g,A}\right)$ and ladybirds $\left(V_{g,L}\right)$ remain unchanged until the next growing season. Aphid and ladybird population size will reduce by 20% due to the overwintering mortality rate and the remaining aphids and ladybirds (80%) are used as the starting values in the next year.

To study the long‐term evolution of the aphids and ladybirds, we first ran the eco‐evolutionary model from 2000 to 2020 to flush the initial conditions and began the simulation from 2021 with realistic starting values. We then ran the three models (Section 2.3.2) separately from 2021 to 2150. We calculated $\int_{1}^{365}Adt$ and $\int_{1}^{365}Ldt$ for each year and denoted them as “annual aphid pressure” (AAP), and “annual ladybird pressure” (ALP). For brevity, we refer them as aphid and ladybird population abundances throughout the paper. By comparing the long‐term patterns of aphids and ladybirds' population abundances in all three models, we can examine the impact of evolutionary adaptation on each species.

#### 2.4.2 Mechanism for geographic variation in evolutionary rescue of predators

Our results demonstrate that evolutionary rescue does not occur everywhere. The predator population abundance is determined by the prey abundance and local climate conditions. So, the extinction of predators could be due to (1) insufficient prey abundance, or (2) inadequate evolutionary response to adapt to unfavorable climatic conditions. To explore the reasons behind (2), we chose two representative locations for comparison, one where evolutionary rescue occurs (SO: Logansport, Louisiana), the other where it does not (NO: Beauford, Minnesota). The climate in the ‘rescued’ location is characterized by warmer temperatures, slower warming trend, and lower seasonality compared to the ‘extinct’ location. At first, we compared the daily prey abundance over time at two locations to verify whether predators go extinct due to insufficient prey. We then investigated the effect of predators' evolutionary potential by adjusting ladybirds' segregation variance $\left(V_{g,L,0}\right)$. We also conducted a sensitivity analysis on aphids' segregation variance $\left(V_{g,A,0}\right)$ to evaluate the model robustness. In addition, we switched the climate parameters, seasonality $\left(s\right)$ and/or warming trend $\left(k\right)$ at the two locations to further explore which climate characteristic has a more important role in determining the possibility of evolutionary rescue for predators.

To further quantitatively compare the effect of the seasonality $\left(s\right)$ and trend $\left(k\right)$, we selected three representative locations (NO, CO, SO) which have the least similar annual mean temperatures ($\bar{T}$) among the nine locations (Table 1). We generated different temperature profiles by varying the values of s and k (Equation 3). By examining the changes in ALP as seasonality and trend increase, we can assess how the degree of evolutionary rescue is impacted by these factors.

## 3 RESULTS

### 3.1 Geographic variation in evolutionary rescue

**FIGURE 3:**
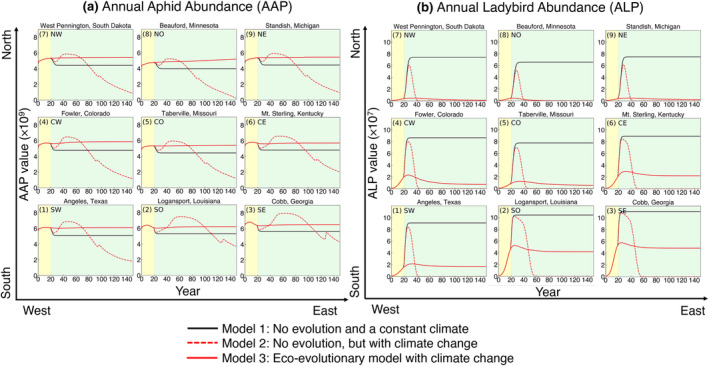
Annual population abundance for aphids (a; AAP) and ladybirds (b; ALP) over 150 years in different locations. Each subplot demonstrates their trends for a single location. We use the first 20 years (yellow zone) to flush the initial condition. The curves with different colors represent the results generated by different models (see legend).

Figure 3 shows the population dynamics for aphids and ladybirds across the nine geographic locations. For the three model scenarios, aphid and ladybird populations have different dynamics over 150 years. In each subplot, the black curve shows the annual population abundances of aphids and ladybirds over time in a stable climate (Model 1). Both species are able to maintain their populations and eventually reach a stable equilibrium. In Model 2, which takes into account climatic change, but no evolutionary response (red dashed curves), the population of aphids declines, while the population of ladybirds goes extinct due to their inability to adapt to the changing climate conditions and/or reduced prey availability. In the eco‐evolutionary model (Model 3, shown by the red solid curves in Figure 3), we find that the mean optimal thermal performance ($T_{\mathrm{opt}}$) of both species increases as a result of natural selection (Figure S1). This adaptation allows the aphid population to persist and maintain its population size (Figure 3a), although abundance varies with location. Ladybirds experience varying degrees of rescue. Southern populations are fully rescued, whereas northern populations are only weakly rescued, or not rescued.

**FIGURE 4:**
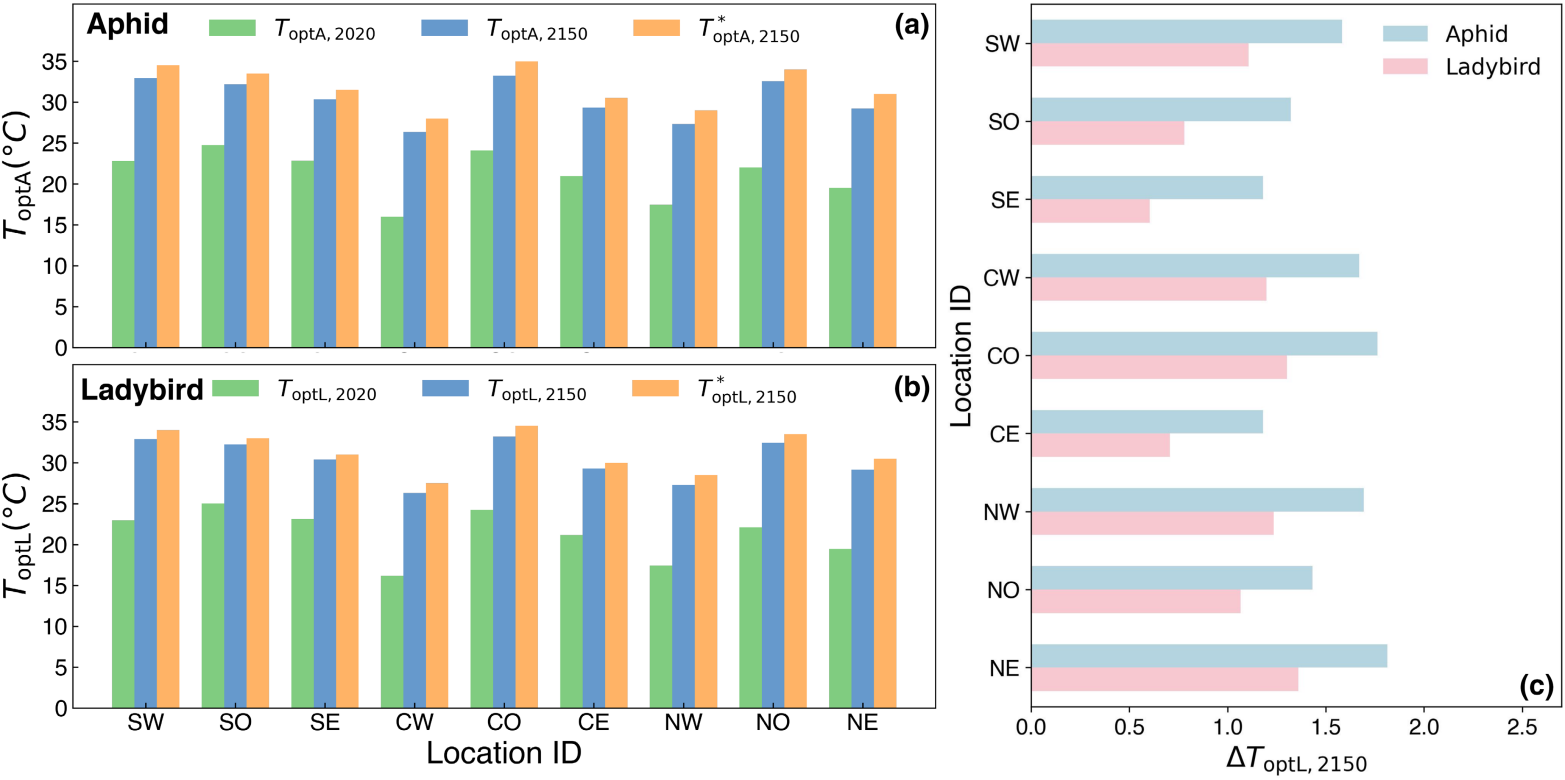
Locally adapted and theoretical $T_{\mathrm{opt}}$ for aphids and ladybirds. The barplots in (a) and (b) compare the $T_{\mathrm{opt}}$ under different conditions across different locations. The green bars and blue bars represent the locally adapted optimal temperatures and in 2020 (aphid: $T_{\text{optA},2020}$; Ladybird: $T_{\text{optL},2020}$) and 2150 (aphid: $T_{\text{optA},2150}$; Ladybird: $T_{\text{optL},2150}$) generated by our eco‐evolutionary model simulations. The orange bars represent the theoretical optimal temperature (aphid: $T_{\text{optA},2150}^{*}$; Ladybird: $T_{\text{optL},2150}^{*}$) that match the local environment in 2150 (as estimated by Supplement S3). (c) shows the differences between $T_{\mathrm{opt},2150}^{*}$ and $T_{\mathrm{opt},2150}$ for both species across different locations.

The barplots in Figure 4a,b show the magnitudes of evolved $T_{\mathrm{opt}}$ for aphids and ladybirds at different locations. The green bars represent the mean phenotypic values of $T_{\mathrm{opt}}$ for aphids and ladybirds in 2020. The blue and orange bars represent the mean phenotypic values of $T_{\mathrm{opt}}$ by 2150; however, the blue bars represent the *realized* phenotypic values estimated by the eco‐evolutionary model, while the orange bars represent the *theoretical* values that match the local environment in 2150 (as estimated by Supplement S3). By comparing the phenotypic values shown in the bar plots, we can see that the evolution of aphids and ladybirds helps both species to keep pace with climate change. This is evident in the increase in their optimal thermal performance $\left(T_{\mathrm{opt}}\right)$ over time. While it is not possible for evolutionary adaptation to keep up with the constantly changing climate due to the inherent time lag, evolution can still compensate for a significant portion of the effects of climate change. In terms of the ability to compensate, ladybirds tend to perform better than aphids. The bigger differences between the theoretical optimum and the evolved optimum for $T_{\mathrm{opt},A}$ compared to $T_{\mathrm{opt},L}$ (Figure 4c) indicates a lower absolute fitness for aphids than ladybirds even when $T_{\mathrm{opt},A}$ and $T_{\mathrm{opt},L}$ evolve at about the same rate (Figure S1). Nevertheless, it is ladybirds that are more weakly rescued.

### 3.2 Is it a lack of prey or evolution that leads a lack of predator rescue?

To explore whether a lack of prey abundance or evolution leads to predator extinction, we first plot the daily population abundance of aphids from 2050 to 2100 at the ‘rescued’ (SO) and ‘extinct’ (NO) locations, respectively (Figure S2a). Surprisingly, the aphid population in the ‘extinct’ location is even larger than in the ‘rescued’ location, so the extinction of the predators is not due to low prey abundance. We then adjusted the segregation variance of ladybirds to demonstrate the effect of additive genetic variance on the population abundances for aphids and ladybirds and their phenotypic values of $T_{\mathrm{opt}}$. As we see in Figure S2b1–d2, a larger $V_{g,L,0}$ allows ladybirds to have a greater evolutionary potential to adapt to climate change and avoid extinction. This suggests that $V_{g,L,0}$, in part, determines the persistence of the ladybird population. Conversely, the sensitivity analysis for aphids ($V_{g,A,0}$, Figure S2e1–g2) indicates that changes in aphid segregation variance minimally impact the evolutionary response of ladybirds. Even with increased $V_{g,A,0}$, ladybirds still face extinction in the ‘extinct’ location due to insufficient evolutionary potential ($V_{g,L,0}$).

**FIGURE 5:**
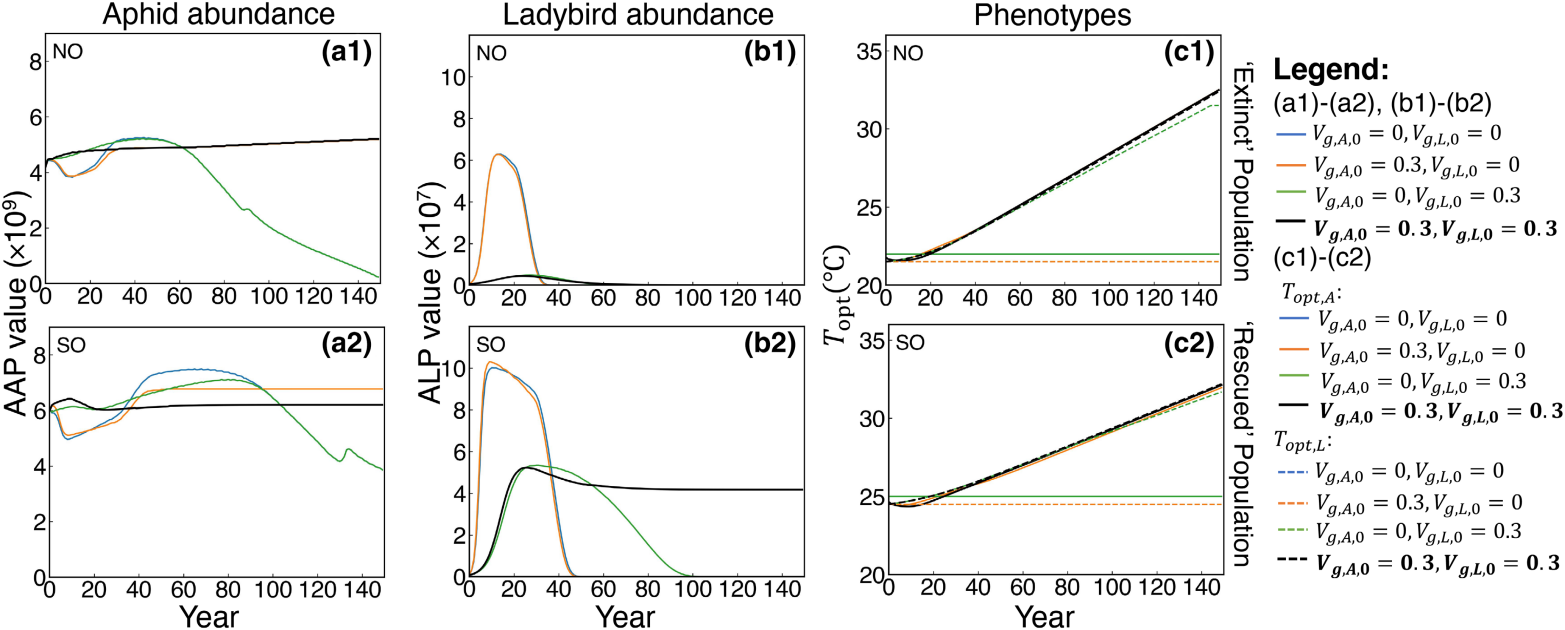
Evolutionary effects on the aphid and ladybird population abundance (AAP and ALP) and their evolving trait $\left(T_{\mathrm{opt}}\right)$ under different evolutionary scenarios in southern (SO: Logansport, Louisiana) and northern (NO: Beauford, Minnesota) locations. Blue curves ($V_{g,A,0}$ = 0 and $V_{g,L,0}$ = 0) represent the baseline scenario without evolution for comparison. Orange curves ($V_{g,A,0}$ = 0.3 and $V_{g,L,0}$ = 0) represent the evolution scenario when only prey evolves. Green curves ($V_{g,A,0}$ = 0 and $V_{g,L,0}$ = 0.3) represent the evolution scenario when only predator evolves. Black curves ($V_{g,A,0}$ = $V_{g,L,0}$ = 0.3) represent the scenario when aphids and ladybirds both evolve with climate change. In (c1) and (c2), solid curves represent $T_{\mathrm{opt},A}$, dashed curves represent $T_{\mathrm{opt},L}$.

Finally, we investigated different evolutionary scenarios for both aphids and ladybirds (Figure 5). If both species cannot evolve by selection ($V_{g,A,0}=V_{g,L,0}=0$, blue curves), both the southern (SO) and northern (NO) populations of ladybirds will go extinct (Figure 5b1,b2) and the size of the aphid population will decrease with climate change (Figure 5a1,a2). If only the prey species (aphids) is able to evolve ($V_{g,A,0}=0.3$, $V_{g,L,0}=0$, orange curves), it will adapt to the changing environment, but ladybirds will still be at risk of extinction, even with this indirect evolutionary effect from aphids. If only the predator species (ladybirds) can evolve ($V_{g,A,0}=0$, $V_{g,L,0}=0.3$, green curve), the aphid population will not be able to adapt to the novel environment and will decrease in size. Due to the lack of sufficient prey, even with the direct evolutionary effect on ladybirds, ladybirds are still not able to be rescued from local extinction. However, when both species evolve in response to climate change ($V_{g,A,0}=V_{g,L,0}=0.3$, black curves), evolution enables aphids to adapt to novel environments and increases the likelihood of rescuing the ladybirds from extinction (Figure 5b1,b2). Overall, these comparisons highlight the importance of evolutionary effects within a predator–prey system.

### 3.3 Is it the trend or seasonality that determines the evolutionary rescue of the predators?

**FIGURE 6:**
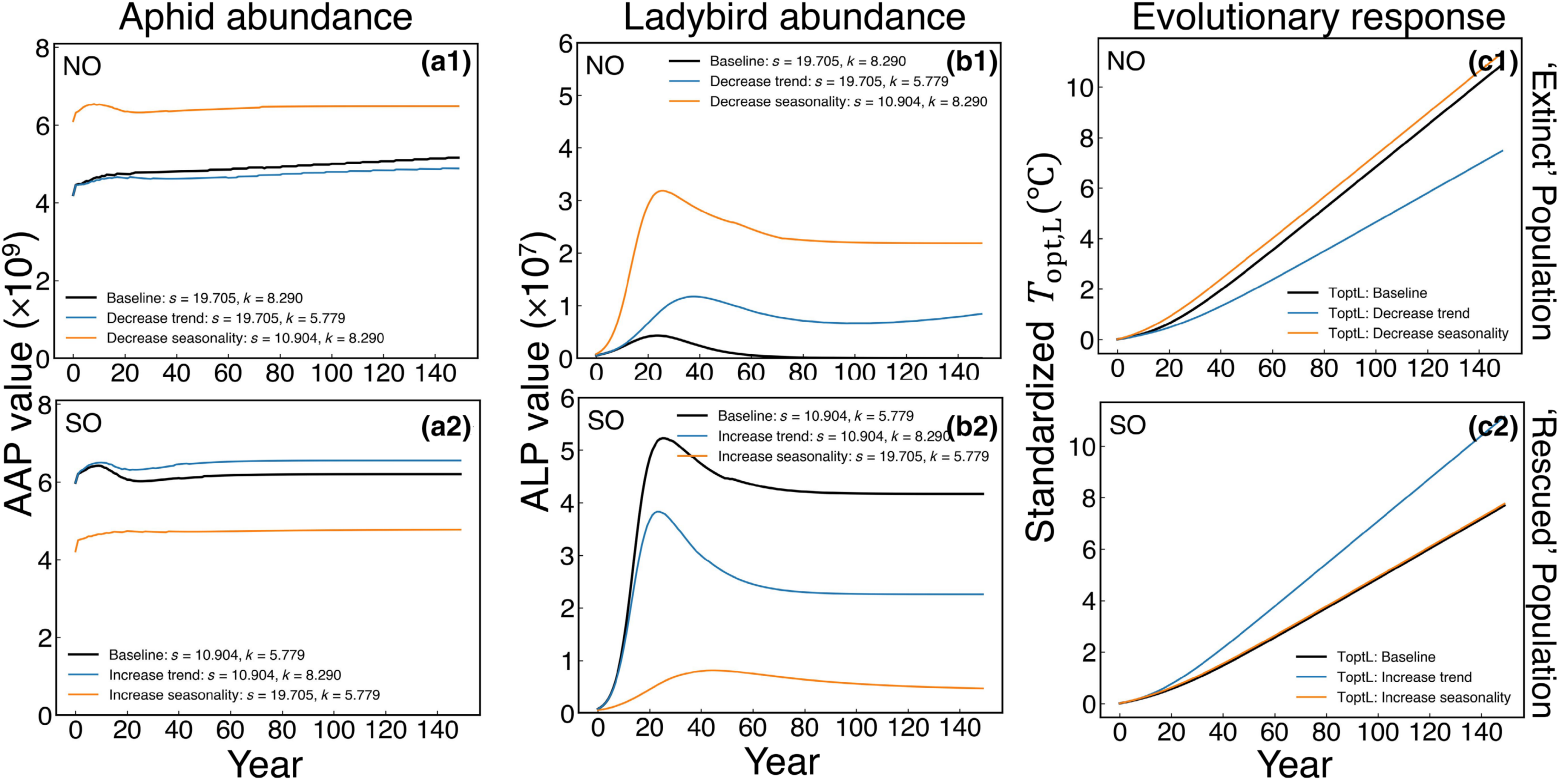
Effect of seasonality $\left(s\right)$ and warming trend $\left(k\right)$ on aphid and ladybird population abundance (AAP and ALP) and their evolving trait $\left(T_{\mathrm{opt}}\right)$ in the ‘rescued’ (SO: Logansport, Louisiana) and the ‘extinct’ (NO: Beauford, Minnesota) locations. The blue curves represent the results generated by using the temperature parameters in each location. We exchanged the temperature parameters (s or k) between Logansport, Louisiana and Beauford, Minnesota to see which parameter is more important. Specifically, we increased the values of s or k for Logansport to the values they have in Beauford, where the climate is more seasonal and has a faster warming trend. Conversely, we decreased the values of s or k for Beauford to the values they have in Logansport. In (c1) and (c2), solid curves represent $T_{\mathrm{opt},A}$, dashed curves represent $T_{\mathrm{opt},L}$. Standardized $T_{\mathrm{opt}}$ values subtract off the initial value of $T_{\mathrm{opt}}$.

We explored whether seasonality $\left(s\right)$ or trend $\left(k\right)$ affects whether a species is ‘rescued’ or goes ‘extinct’ by manipulating these climate parameters for a given location. As shown in Figure 6b1,b2, we switch the s and k between the ‘rescued’ and ‘extinct’ locations, and find that lower seasonality or slower warming trend both lead to an evolutionary rescue in the ladybird population or a higher ladybird population abundance. Moreover, by gradually increasing s and k in three locations (SO, CO, NO) with different annual mean temperatures ($\bar{T}$) and plotting ladybird population abundances (ALP) in 2150 under various climate conditions, we find a consistent pattern (Figure S3) that aligns with the findings shown in Figure 6. Our results suggest that an increase in both of the two climate parameters has negative effects on the degree of evolutionary rescue of ladybird populations. However, it is important to note that the mechanisms by which they affect evolutionary rescue are different, as described next.

As we decrease or increase k (Figure 6c1,c2, blue curves), we see the rate of change in standardized $T_{\mathrm{opt},L}$ (i.e., $T_{\mathrm{opt},L}-T_{\mathrm{opt},L,2000}^{*}$) becomes lower (flatter curve) or higher (steeper curve), corresponding to a weaker or stronger evolutionary response. In the ‘extinct’ location (NO), evolutionary adaptation occurs fast enough to keep pace with a slowed warming trend (Figure 6b1–c1, blue curve). In contrast, an increased warming trend in the ‘rescued’ location (SO) causes ladybirds to undergo an unfavorable hotter summer, which leads to a lower abundance even with a strong evolutionary response (Figure 6b2–c2, blue curve). For a given seasonality, a faster warming trend results in a reduced population abundance or extinction for ladybirds (Figure S3). However, the evolutionary compensation exhibited by ladybirds in response to the rate of climate change mitigates this effect, resulting in a relatively smaller population change when compared to the impact of seasonality.

Seasonality does not affect the evolutionary response of ladybirds (similar slopes between the black and orange curves Figure 6c1,c2). However, a higher seasonality still leads to a reduced population abundance of ladybirds (Figure 6b2, black‐orange curve) or even the possibility of their extinction (Figure 6b1, orange‐black curve). As the climate becomes more seasonal, the reproductive season of ladybirds tends to be shorter, which leads to a lower ladybird population abundance within a year (Figure S4). Subsequently, the ladybirds remain at a lower population abundance or go extinct by 2150 (Figure S3). This indicates that higher seasonality reduces the capacity for evolutionary rescue by limiting the length of the reproductive season.

To summarize, our findings demonstrate that both seasonality and warming trend play significant roles in determining the degree of evolutionary rescue in predators. The impact of seasonality primarily arises from ecological processes, while the effect of the warming trend is influenced by a combination of ecological and evolutionary processes. While evolutionary adaptation serves as the fundamental driver of evolutionary rescue, it is crucial to recognize the importance of ecological processes and the complex interplay between the two aspects.

## 4 DISCUSSION

Our findings suggest that there is geographic variation in thermal performance evolution and evolutionary rescue for ladybirds across different locations east of Rocky Mountains in the United States. As climates become cooler and more seasonal, the degree of evolutionary rescue becomes weaker. Ladybirds can be rescued from extinction in most regions, but may still face extinction in the north, unlike aphids, their prey. The primary cause of ladybird extinction in these regions is the insufficient evolutionary potential to adapt to climate changes (seasonality and warming trend). Notably, our results indicate that interacting species respond to the impacts of seasonality and warming trends through ecological and eco‐evolutionary processes, respectively. This reveals the essential role of ecological processes in predicting evolutionary adaptation outcomes. These findings highlight the importance of understanding the complex interplay between ecological and evolutionary processes in determining evolutionary rescue and species habitat suitability.

Current research on the spatial patterns of evolutionary potential primarily examines individual species. For an insect pest, which is regulated by both bottom‐up effect and top‐down control, it is essential to understand how the interacting species respond to climate change (Chidawanyika et al., 2019). Our model indicates that ladybirds are less likely to be rescued by evolutionary adaptation. Accordingly, aphids may lose a natural predator because of differences in evolutionary rescue, with secondary impacts on crop yields and host‐pathogen dynamics in the case of plant–viruses interactions. Apart from the predator–prey interactions, intraspecific and interspecific competition can have negative and positive effects on the possibility of evolutionary rescue (De Mazancourt et al., 2008; Osmond & De Mazancourt, 2013). Intraspecific competition tends to hinder evolutionary rescue by lowering mutation rates due to reduced populations. In contrast, interspecific competition can promote evolutionary rescue by enhancing selection pressure (Osmond & De Mazancourt, 2013). Additionally, the presence of multiple species often means some are pre‐adapted to new conditions, limiting the opportunity for other species to evolve in response to environmental changes (De Mazancourt et al., 2008). The complex interplay among different types of biotic interactions underscores the need for increased study on the evolutionary dynamics of interacting species (Fussmann et al., 2007). Such research may provide valuable insights to enhance pest management strategies.

Geographic variation in evolutionary rescue for ladybirds reveals a synergistic mechanism connecting ecological and evolutionary responses in predators, which may have important implications for agriculture. Our findings indicate that a predator is more likely to be rescued in the southeastern locations. In these regions, climates tend to be more stable and warm slower (Table 1). From an evolutionary perspective, ladybirds can more easily adapt to relatively slower climate change, thus improving their chances of survival in these areas. From an ecological perspective, these climates allow both aphids and ladybirds to experience a long reproductive season, ultimately beneficial for maintaining larger populations. Altogether, ladybirds can adapt to the changing climate through a combination of a long reproductive season, sufficient prey, and evolution. In contrast, in the northwestern regions where climates are more seasonal and experience rapid temperature increases, both species have a shorter reproductive season, affecting their population size and may further limit the food availability for the predator. A key finding is that although the evolutionary response of ladybirds was strong, equaling or better than aphids, it went extinct in northern locations. This result points to the interplay between evolutionary response and population ecology (predator–prey interactions) in assessing the potential for evolutionary rescue.

In our system, if only the prey evolves in response to climate change, predators will not be rescued. “Indirect evolutionary rescue” (IER) mechanism does not seem to occur, whereby a non‐evolving predator can be rescued from extinction solely due to the evolution of its prey (e.g., Yamamichi & Miner, 2015). The IER mechanism assumes the defense cost for prey against predation relies on predator density. If predators are scarce, prey defense is reduced, which indirectly results in an increased population growth rate of the predators (Yamamichi & Miner, 2015). Our study did not include the fitness cost of prey defense, instead focusing on the thermal effects on species' vital rates. Even if the prey adapts to the changing climate and provides sufficient food resources for predators, predators may still face extinction as their thermal performances do not match the novel climate. This indicates evolution of both prey and predator supports predator persistence. Despite the inconsistency in these findings, our research, along with the IER mechanism, provides a theoretical basis for understanding the predator–prey interaction effects on evolutionary responses under climate change from different perspectives.

Simplifications in our model include modeling only a horizontal shift in thermal performance. Previous research has outlined three ways in which thermal performance curves (TPCs) can change through evolution or plasticity: (1) variation in overall performance (vertical shift); (2) variation in the thermal optimum (horizontal shift) and (3) variation in thermal specialization (Sinclair et al., 2012, their Figure 1). These variations can also occur within geographic gradients (MacLean et al., 2019; Tüzün & Stoks, 2018). In general, we can incorporate the multiple dimensions of TPCs evolution by making $z_{A}$ and $z_{L}$ multivariate, then integrate across multiple dimensions in a model that considers additive covariance among these dimensions. However, we focus on a horizontal shift as a first step.

The climate change patterns used in this study are also simplified. We assume only the warming trend leads to the increase in daily temperatures, while seasonality remains unchanged over time. However, climate projections indicate that most land area across the world will experience an increase in seasonality over time (Ge et al., 2023). As indicated in our study, seasonality may lead to a quantitative shift in the population abundance of predators. Going forward, it is worthwhile to explore how species adapt to various climatic changes by accounting for interactions between the warming trend and changing seasonality.

Additional limitations of our eco‐evolutionary model include the lack of phenotypic plasticity and dispersal. Plasticity in $T_{\mathrm{opt}}$, ${\mathrm{CT}}_{\min}$, and ${\mathrm{CT}}_{\max}$ may also play a role in driving genetic variation and can affect the rate of genetic change, either by slowing it down or accelerating it (Kopp & Matuszewski, 2014). A recent model of adaptation to shifting environments with phenotypic plasticity conducted individual‐based simulations to demonstrate how tolerance curves may evolve and affect species' adaptation and highlights the spatial variation in plasticity (Schmid et al., 2019). However, the lack of empirical evidence regarding spatial heterogeneity and genetic variation in species traits, as well as the complex mechanisms underlying spatial differences in tolerance and plasticity, has compounded the challenges of integrating plasticity into our model (Schmid et al., 2019). Future research should extend and enrich the exploration of plasticity mechanisms, taking into account various factors influencing the spatial and temporal variations in phenotypic plasticity. This fundamental research can lay the theoretical foundation for incorporating other sources of phenotypic plasticity to assess their relative importance in evolutionary rescue. Dispersal can influence how evolution affects species' range shifts under climate change; it mitigates the negative effects of climate change and accelerates climate‐induced range expansion as highly mobile individuals may be able to track suitable climates (Akesson et al., 2021; Nadeau & Urban, 2019). While some recent research has taken into account the role of dispersal in predicting species range shifts, they have primarily focused on individual species, rather than multiple interacting species (Bush et al., 2016; DeMarche et al., 2019; Diniz‐Filho et al., 2019). Our model serves as a baseline controlling for dispersal. To include dispersal in the paper's modeling framework requires consideration of what is the appropriate scale to model subpopulations. For example, is it informative to model dispersal between a Georgia and a Michigan population, or is a finer scale or diffusion model needed? Secondly, dispersal's effects on genetic variance and trait mean would need to be model correctly (e.g., Guillaume & Whitlock, 2007).

Despite these limitations, our study accounts for changes in genetic variance caused by natural selection using Bulmer's (1971) infinitesimal model. Our paper presents an approach to numerically implementing Bulmer's model, while also accounting for population dynamics. In addition, our model provides informative predictions. Taking evolutionary adaptation into account, the agricultural pest can be rescued, however, their predators may experience varying degrees of evolutionary rescue based on region. Seasonality and warming trend in different locations affect the degree of evolutionary rescue through ecological and evolutionary processes. Overall, our study supports the advancement of SDMs in the area of evolution and species interactions within agricultural systems, offering causal insight into variation in evolutionary rescue of a predator to a crop pest.

## CONFLICT OF INTEREST STATEMENT

We have no conflict of interest to declare.

## Supporting information


Data S1.

## Data Availability

The model code that supports the findings of this study is openly available in: https://zenodo.org/records/12244251.
