# Supplementary material for: Geographic variation in evolutionary rescue under climate change in a crop pest–predator system
Source: Evol Appl. 2024 Jul 22;17(7):e13750. doi: 10.1111/eva.13750 (PMC11261214; doi:10.1111/eva.13750)
Supplement: Supplementary file 1 — Data S1. [file EVA-17-e13750-s001.pdf]

ORIGINAL ARTICLE

Geographic variation in evolutionary rescue under climate change in a crop pest–predator system

Xuezhen Ge[1,2*] | Jonathan A. Newman[2] | Cortland K. Griswold[1]

[1] Department of Integrative Biology, University of Guelph, Guelph, N1G 2W1, Canada
[2] Department of Biology, Wilfrid Laurier University, Waterloo, Ontario, N2L 3C5, Canada
* Correspondence: Xuezhen Ge, Current address: Department of Biology, Wilfrid Laurier University, Waterloo, Ontario, N2L 3C5, Canada, xuezhen.ge@gmail.com

1 | SUPPORTING FIGURES

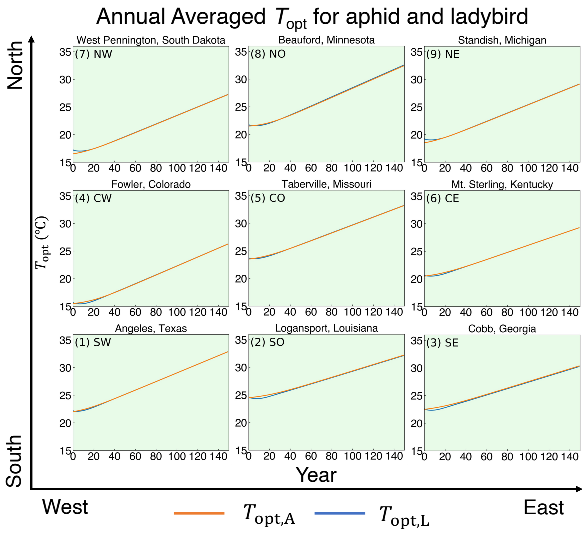

FIGURE S1 Changing trend of annual averaged T_{opt} for aphid ($T_{opt,A}$, blue curve) and ladybird $T_{opt,L}$, orange curve) over 150 years.

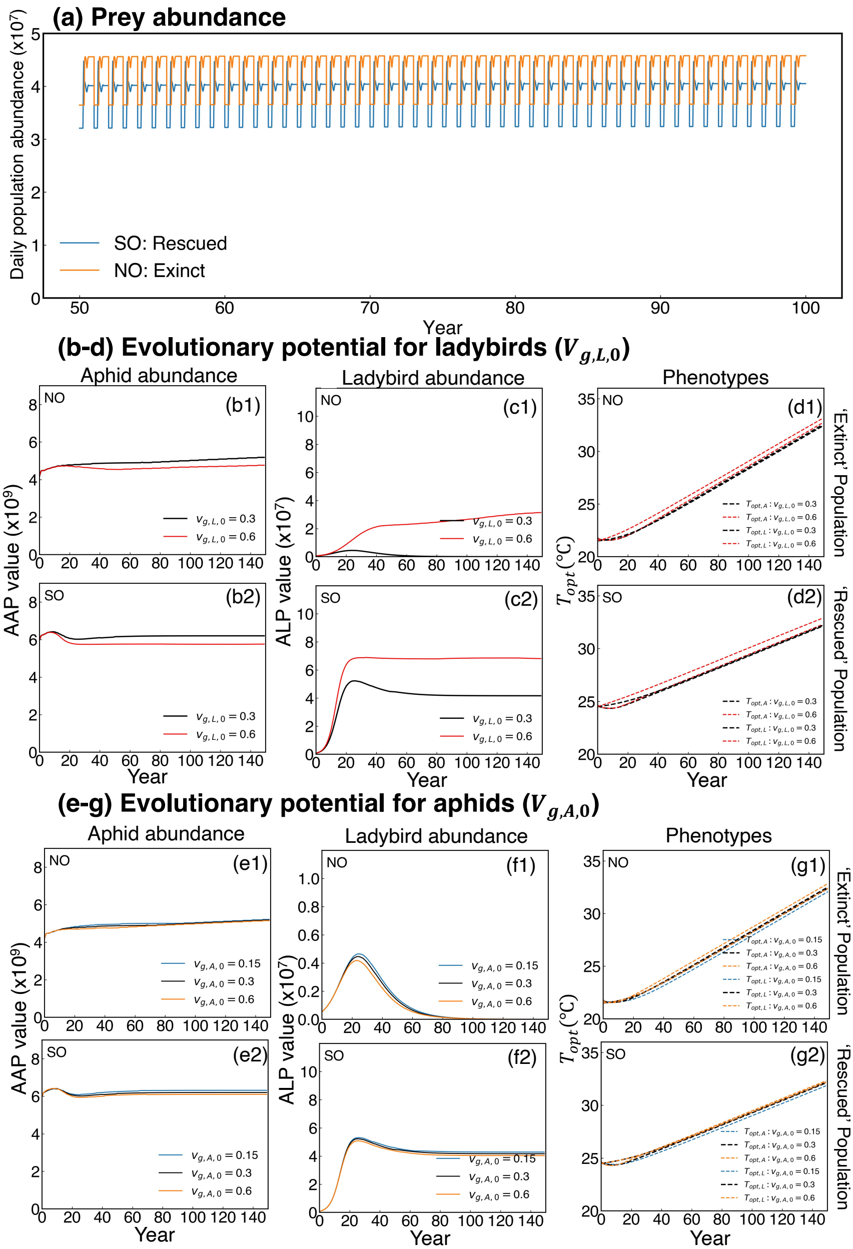

FIGURE S2 Daily aphid population abundance and effect of evolutionary potential on aphid and ladybird population abundance (AAP and ALP) and their evolving trait, T_{opt}, in the 'rescued' (SO: Logansport, Louisiana) and 'extinct' (NO: Beauford, Minnesota) locations. (a) daily population abundance for aphids from 2050-2100 in the two locations. (b1)-(d2) represent the changing trend for AAP, ALP and T_{opt} when altering ladybirds' segregration variance (Black curves:$V_{g,L,0}$=0.3; Red curves:$V_{g,L,0}$=3). (e1-g2) represent the changing trend for AAP, ALP and T_{opt} when altering aphids' segegration variance (Blue curves: $V_{g,A,0}$ = 0.15, Black curves:$V_{g,A,0}$=0.3; Red curves:$V_{g,A,0}$=3) for ladybirds. In (d1), (d2), (g1) and (g2), solid curves represent $T_{\text{opt},A}$, dashed curves represent $T_{\text{opt},L}$.

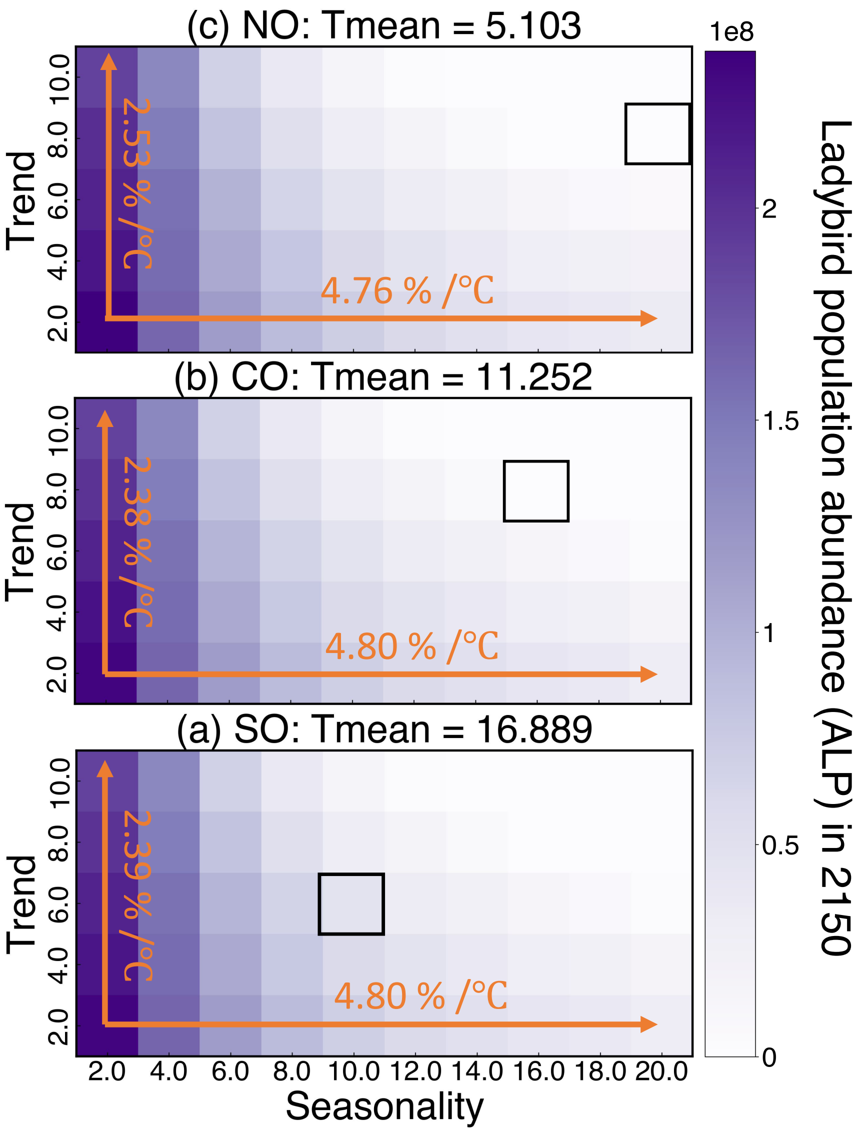

FIGURE S3 Ladybird population abundance (ALP) in 2150 under different climates with various seasonality (s) and warming trend (k). (a)-(c) represent three different locations (SO: Logansport, Louisiana; CO: Taberville, Missouri; NO: Beauford, Minnesota). The color depth in the heatmap indicates the magnitude of ALP, which also represents the degree of evolutionary rescue. A lighter color indicates weaker evolutionary rescue. The black box in each subplot indicates the actual combination of seasonality and trend in each location. The orange numbers in each subplot denote the rate of reduction in ladybird population abundance as we increase seasonality or trend.

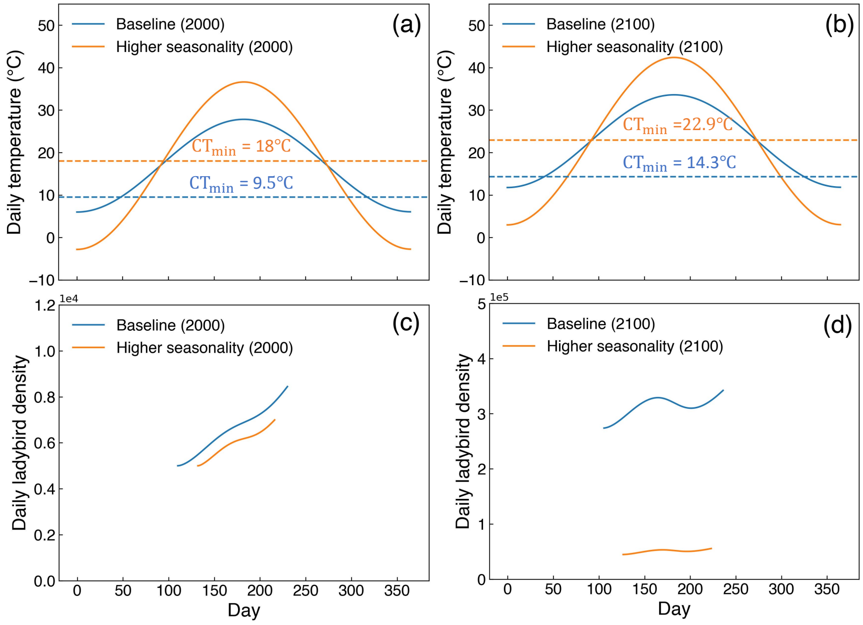

FIGURE S4 Daily temperatures and ladybird density in 2000 and 2100 in Louisiana (SO). 'Baseline' (blue curves) means we use the actual seasonality value in Louisiana. 'Higher seasonality' (orange curves) represents that we use the seasonality value in Minnesota (NO), which has a much higher seasonality. (a)-(b) daily temperature in 2000 and 2100. (c)-(d) daily ladybird density in 2000 and 2100. We only plot the reproductive season in (c) and (d).

2 | SUPPORTING TABLES

TABLE S1 Notations and parameters in the eco-evolutionary model.

Notation	Definition and Parameter value	Note
T_t	Daily mean temperature	Eq. 1
$\bar{T}$	Initial annual mean temperature in 2000 in each location.	Eq. 1
s	Seasonality, defined as half of the difference between yearly minimum temperature $T_{min,year}$ and yearly maximum temperature $T_{max,year}$	Eq. 2
k	Warming trend, defined as the change of annual mean temperature every century in each location	Eq. 3
DL	Daylength, the required number of daylight hours, is determined by ξ, which is the exposed radius between the sun's zenith and the sun's solar circle and estimated by a function of latitude (ϕ), the number of days from January 1st (t) and the Earth's rotational axis $R = 23.439°$	Eqs. 36
ε	A computational control "switch" to enable the sexual phase, aphids remain in their asexual phase when $\varepsilon = 0$ and switch to their sexual phase when $\varepsilon = 1$.	Eq. 36
A, L	Population density of aphids or ladybirds	-
AAP, ALP	Accumulation of the daily population abundance for aphids or ladybirds within each year	-
$z_{A,i}$, $z_{L,j}$	Phenotypic value of optimumal temperature T_{opt} for an aphid individual or ladybird individual.	Eqs. 4-5
$z_{g,A,i}$, $z_{g,L,j}$	Genotypic value of an aphid individual or ladybird individual	Eqs. 4-5
$z_{e,A,i}$, $z_{e,L,j}$	Environmental effects on the phenotype of an aphid individual or ladybird individual.	Eqs. 4-5

Continued on next page

Table S1 – continued from previous page

Notation	Definition and Parameter value	Note
$\bar{z}_{g,A}$, $\bar{z}_{g,L}$	The mean breeding value for the population of aphids or ladybirds.	-
$\bar{z}_A$, $\bar{z}_L$	The mean phenotypes for the population of aphids or ladybirds	-
$\bar{z}^*_A$, $\bar{z}^*_L$	Locally adapted optimal temperature for aphids or ladybird	-
$\{z_{A,i}\}$, $\{z_{L,j}\}$	Continuous set of phenotypes in the population for aphids or ladybirds, respectively.	Eq. 6
$f(z_{A,i}, T, t)$	The birth rate for an aphid individual at time t.	Eq. 6
$\bar{f}(\{z_{A,i}\}, T, t)$	The mean birth rate for the aphid population at time t.	Eq. 6
$\mu(z_{A,i}, T, t)$, $\gamma(z_{L,j}, T, t)$	The intrinsic mortality rates for an aphid individual or ladybird individual at time t, respectively.	Eq. 6
$\bar{\mu}(\{z_{A,i}\}, T, t)$, $\bar{\gamma}(\{z_{L,j}\}, T, t)$	The mean intrinsic mortality rates for aphids or ladybirds at time t, respectively.	Eq. 6
$g(z_{A,i}, T, t)$	The effect of temperature on the functional response, range from 0–1.	Eq. 8
$w_A(z_{A,i}, z_{L,j}, t)$	The instantaneous rate of growth of the descendant lineage for an aphid with phenotype $z_{A,i}$ at time t.	Eq. 9
$w_L(z_{L,j}, t)$	The instantaneous rate of growth of the descendant lineage for a ladybird with phenotype $z_{L,j}$ at time t.	Eq. 11
$\rho_{(z_{g,A},t)}$, $\rho_{(z_{g,L},t)}$	The probability densities of aphid or ladybird individuals with genotype $z_{g,A}$ or $z_{g,L}$.	Eqs. 21-22
$\rho_{(z_{e,A},t)}$, $\rho_{(z_{e,L},t)}$	The probability densities of aphid or ladybird individuals with environmental effect $z_{e,A}$ or $z_{e,L}$.	Eqs. 21-22
ρ_τ	The probability density for the mutational effect of aphids.	Eq. 30
$V_{g,A}$, $V_{g,L}$	Additive genetic variance for aphids or ladybirds. Segregation variance $V_{g,A,0}$ = $V_{g,L,0}$ = 0.3	-

Continued on next page

Table S1 – continued from previous page

Notation	Definition and Parameter value	Note
$V_{e,A}$, $V_{e,L}$	Environmental variances for aphids and ladybirds. $V_{e,A}$ = $V_{e,L}$ = 0.7	-
f	Inbreeding coefficient for the aphid population. f = 0.1	Eq.5
$h^2_{m,s}$	Mutational heritability for aphids ($s = A$) and ladybirds ($s = L$). We selected 0.002 (estimated for the thermal performance of *Drosophila*) as the approximate estimate for $h^2_{m,A}$ and $h^2_{m,L}$ (Houle et al., 1996; Lynch and Walsh, 1998; Latimer et al., 2014).	-
η_s	Generation time coefficient for aphids ($s = A$) and ladybirds ($s = L$). The rough estimates for η_A and η_L are 0.05 generations/half-day and 0.01 generations/half-day, respectively.	-
$V_{\tau,s}$	Mutational variance for aphids $s = A$ and ladybirds ($s = L$). $V_{\tau,s} = h^2_{m,s} \times V_{e,s} \times \eta_s$.	Eqs. 25, 34
K	Carrying capacity for aphid population.	Eq. 6
m_A	Optimal growth rate at optimum temperature, its value depends on temperature-dependent generation time. m_A = 0.6	Eq. S1
$\mathrm{CT}_{\mathrm{min}}$, $\mathrm{CT}_{\mathrm{max}}$	Minimum or maximum temperature thresholds beyond which growth rate is nil, mortality rate is maximal. $\mathrm{CT}_{\mathrm{min}} = z_{A,i}/z_{L,j} - 15$, $\mathrm{CT}_{\mathrm{max}} = z_{A,i}/z_{L,j} + 10$.	Eq. S1
$\mathrm{CT}_{\mathrm{opt1}}$,$\mathrm{CT}_{\mathrm{opt2}}$	Temperature range in which mortality is minimal. $\mathrm{CT}_{\mathrm{opt1}} = z_{A,i}/z_{L,j} - 5$, $\mathrm{CT}_{\mathrm{opt2}} = z_{A,i}/z_{L,j} + 5$	Eq. S3
q_1, q_2	Shape parameters used to adjust the skewness of the thermal performance curve. $q_1 = 1.5$, $q_2 = 1$	Eq. S1
k_1, b_1, k_2, b_2	Shape parameters used to estimate the intrinsic mortality rate for aphids or ladybirds. For aphids, $k_1 = -0.02$, $b_1 = 0.45$, $k_2 = 0.04$, $b_2 = -1.15$. For ladybirds, $k_1 = -0.009$, $b_1 = 0.19$, $k_2 = 0.018$, $b_2 = -0.53$.	Eq. S2

Continued on next page

Table S1 – continued from previous page

Notation	Definition and Parameter value	Note
υ_{min}, υ_{max}	Minimum and maximum mortality rate for aphids or ladybirds. For aphids, υ_{min} = 0.05, υ_{max} = 0.25. For ladybirds, υ_{min} = 0.01, υ_{max} = 0.1.	Eq. S2
a	Searching time for the ladybird to encounter an aphid. a = 0.000001	Eq. 8
h	Handing time for the ladybird to process an aphid. h = 0.000001	Eq. 8
Q_p	Transformation rate, denotes the mean number of aphids a ladybird beetle offspring needs during its generation time, its value depends on the temperature-dependent generation time. 500~2000. Q_p^* = 500 and Q_p' = 2000 represent the minimum and maximum values for Q_p	Eq.s 7, S3

3 | SUPPORTING DOCUMENTS

3.1 | Supplement S1. Parameters for temperature-dependent vital rates.

In our model, all of the thermal traits (e.g., CT_{min} and CT_{max}) for aphids and ladybirds are assumed to evolve with climate change at the same rate with T_{opt}, so they can be expressed as functions of $z_{A,i}$ or $z_{L,j}$ and time t. $z_{A,i}$ or $z_{L,j}$ represents the phenotype of optimal temperature for each individual aphid or ladybird, with considering the genotypical and environmental effects. By incorporating these evolving thermal traits, we expand upon the two temperature-dependent functions within the stage-structured population dynamic model previously developed by Ge et al. (2023) to estimate the vital rate at time t for individual aphid or ladybird. The functions for an aphid individual

with phenotype $z_{A,i}$ are

$$g(z_{A,i},T,t)=\begin{cases} m_A\frac{(T-\mathrm{CT_{min}}(z_{A,i},t))^{q_1}(\mathrm{CT_{max}}(z_{A,i},t)-T)^{q_2}}{(z_{A,i}-\mathrm{CT_{min}}(z_{A,i},t))^{q_1}(\mathrm{CT_{max}}(z_{A,i},t)-z_{A,i})^{q_2}} & \text{if } \mathrm{CT_{min}}(z_{A,i},t)\le T\le \mathrm{CT_{max}}(z_{A,i},t) \\ 0 & \text{otherwise} \end{cases}, \tag{S1}$$

$$\upsilon(z_{A,i},T,t)=\begin{cases} k_1(T-(z_{A,i}-z_{\mathrm{base}}))+b_1 & \text{if } \mathrm{CT_{min}}(z_{A,i},t)\le T\le \mathrm{CT_{opt1}}(z_{A,i},t) \\ \upsilon_{\min} & \text{if } \mathrm{CT_{opt1}}(z_{A,i},t)< T\le \mathrm{CT_{opt2}}(z_{A,i},t) \\ k_2(T-(z_{A,i}-z_{\mathrm{base}}))+b_2 & \text{if } \mathrm{CT_{opt2}}(z_{A,i},t)< T\le \mathrm{CT_{max}}(z_{A,i},t) \\ \upsilon_{\max} & \text{otherwise} \end{cases}. \tag{S2}$$

The functions for a ladybird individual with phenotype $z_{L,j}$ are the same, but with different parameter values. In Eq. S1, $g(z_{A,i},T,t)$ is used to denote the temperature-dependent birth rate for an aphid individual at time t, $g(z_{L,j},T,t)$ denotes temperature effect on the predation rate and birth rate of a ladybird individual at time t. In Eq. S2, $\upsilon(z_{A,i},T,t)$ and $\upsilon(z_{L,j},T,t)$ are utilized to represent the intrinsic mortality rate for an aphid individual and ladybird individual, respectively. T is the air temperature. $\mathrm{CT_{min}}$ and $\mathrm{CT_{max}}$ are the minimum and maximum temperature thresholds beyond which birth rate (or predation rate) is nil, or mortality rate is maximum. $\mathrm{CT_{opt1}}$ and $\mathrm{CT_{opt2}}$ define the temperature range in which mortality is minimal. q_1 and q_2 are shape parameters which adjust the skewness of the thermal performance curve. k_1, b_1, k_2 and b_2 are the parameters which make $\upsilon(\mathrm{CT_{opt1}})=\upsilon(\mathrm{CT_{opt2}})=\upsilon_{max}$ and $\upsilon(\mathrm{CT_{min}})=\upsilon(\mathrm{CT_{max}})=\upsilon_{min}$. We hold $q_1=1.5$ and $q_2=1$ constant, and set $\mathrm{CT_{min}}$, $\mathrm{CT_{max}}$, $\mathrm{CT_{opt1}}$ and $\mathrm{CT_{opt2}}$ as functions of T_{opt}, to ensure the thermal performance curves have the same shape over time. For $g(z_{A,i},T,t)$ and in the context of fecundity, m is set to be $m_A=0.6$ to roughly match the maximum intrinsic rate of growth of aphid ($r_{mA}=0.526$) based on the empirical data under its optimal temperature (Satar et al., 2005).

The impact of temperature on the predation rate of ladybirds is already accounted for in Eq. 8, which uses $g(z_{L,j},T,t)$ to adjust the functional response of ladybirds, setting $m_L=1$. In addition, the temperature effect on the birth rate of ladybirds is represented through the transformation rate Q_p (i.e., the mean number of aphids a ladybird needs to consume to reproduce a single egg), which determines the numerical response of ladybirds. Thus, the

temperature-dependent Q_p for each individual ladybird with phenotype $z_{L,j}$ is

$$Q_p(z_{L,j}, T, t) = \begin{cases} \min(\frac{Q_p^*}{g(\bar{z}, T)}, Q_p') & \text{if } \mathrm{CT}_{\min}(z_{L,j}, t) \leq T \leq \mathrm{CT}_{\max}(z_{L,j}, t) \\ Q_p' & \text{otherwise} \end{cases}, \quad \text{(S3)}$$

where $Q_p^* = 500$ and $Q_p' = 2000$ represent the minimum and maximum values for Q_p which ensures the range of the intrinsic rate of growth for ladybirds (r_{mL} : 0 – 0.2) matches with empirical data under ideal condition (i.e., prey saturation and no competition) (Islam et al., 2022). In our case, r_{mL} is typically in the range of 0 – 0.02 given the carrying capacity of aphids in our model (Fig. S5).

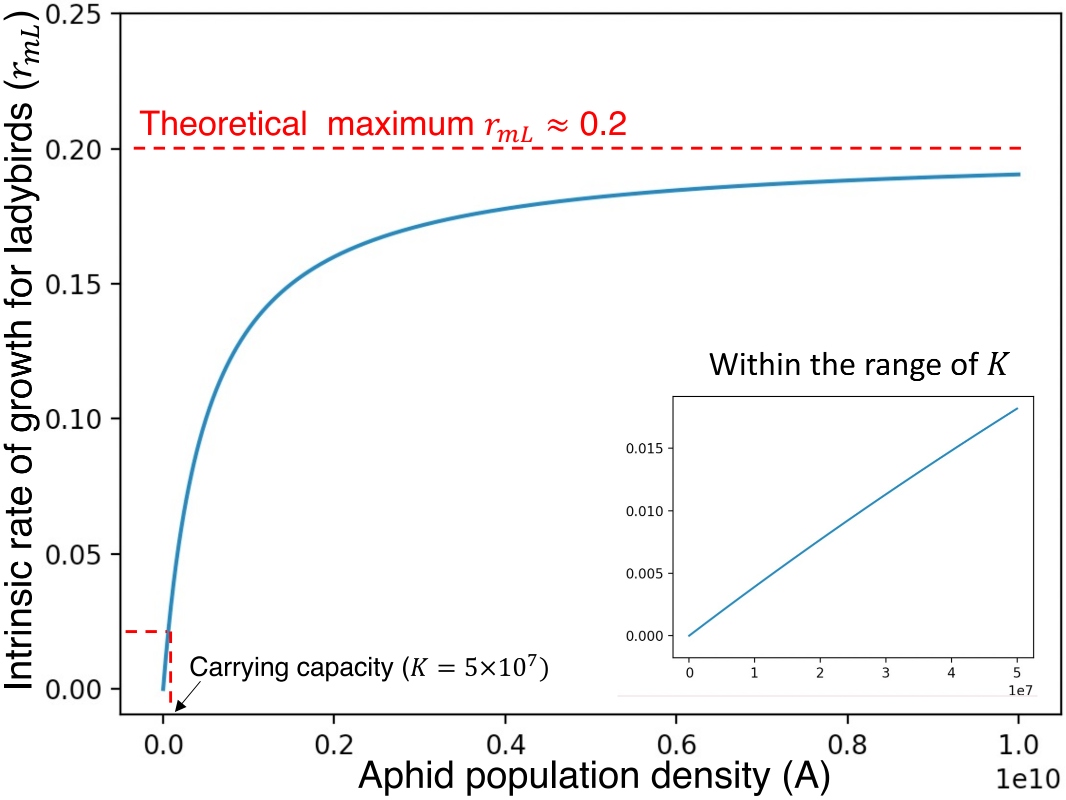

FIGURE S5 Intrinsic rate of growth for ladybirds changes with aphid population density.

3.2 | Supplement S2. Mathematical derivations.

For the aphid population, the un-normalized probability density of each aphid genotype is

$$\begin{aligned}\tilde{\rho}(z_{g,A}, t+\Delta t) = \iiint & \rho(z_{g,A}, t) + (F(z_{g,A} + z_{e,A}, t) - B(z_{g,A} + z_{e,A}, t)) \\ & \rho(z_{g,A}, t)\Delta t\ \rho(z_{e,A}, t)\rho(z_{g,L}, t)\rho(z_{e,L}, t))\ \mathrm{d}z_{e,A}\mathrm{d}z_{g,L}\mathrm{d}z_{e,L}\end{aligned} \tag{S4}$$

$$= \rho(z_{g,A}, t) + \int F(z_{g,A,t} + z_{e,A,t}, t)\rho(z_{g,A}, t)\rho(z_{e,A}, t)\Delta t \mathrm{d}z_{e,A} - \rho(z_{g,A}, t)\Delta t \bar{B}_L(t) \tag{S5}$$

$$= \rho(z_{g,A}, t)(1 + \Delta t \int F(z_{g,A,t} + z_{e,A,t}, t)\rho(z_{e,A}, t)\ \mathrm{d}z_{e,A} - \Delta t \bar{B}_L(t)). \tag{S6}$$

As noted in Eq. 16 in main text, the finite population growth rate for the aphid population is $1 + (\bar{F}_A(t) - \bar{B}_L(t))\Delta t$, thus, the normalized format $\rho(z_{g,A}, t + \Delta t)$ is

$$\rho(z_{g,A}, t+\Delta t) = \frac{\rho(z_{g,A}, t)(1 + \Delta t \int F(z_{g,A,t} + z_{e,A,t}, t)\rho(z_{e,A}, t)\ \mathrm{d}z_{e,A} - \Delta t \bar{B}_L(t))}{1 + (\bar{F}_A(t) - \bar{B}_L(t))\Delta t}. \tag{S7}$$

Similar to the aphid, the un-normalized probability density of each ladybird genotype is

$$\begin{aligned}\rho(z_{g,L}, t+\Delta t) &= \int \rho(z_{g,L}) + w_L(z_{g,L} + z_{e,L,t}, t)\rho(z_{g,L}, t)\rho(z_{e,L}, t))\ \Delta t\ \mathrm{d}z_{e,L} \\ &= \rho(z_{g,L}, t)(1 + \Delta t \int w_L(z_{g,L} + z_{e,L}, t)\rho(z_{e,L}, t)\ \mathrm{d}z_{e,L}),\end{aligned} \tag{S8}$$

then, the normalized $P(z_{g,L}, t + \Delta t)$ is

$$\rho(z_{g,L}, t + \Delta t) = \frac{\rho(z_{g,L}, t)(1 + \Delta t \int w_L(z_{g,L} + z_{e,L}, t)\rho(z_{e,L}, t)\ \mathrm{d}z_{e,L})}{1 + \bar{w}_L(t)\Delta t}. \tag{S9}$$

3.3 | Supplement S3. Locally adapted optimum temperature for aphids and ladybirds

To model the population dynamics of aphids and ladybirds in various geographic locations, we used daily temperature data in 2000 to estimate the locally adapted optimal temperature for each species ($\bar{z}_A^*$ for aphids and $\bar{z}_L^*$ for ladybirds). These estimates are used as the starting values for our long-term simulations. We followed the procedures below to estimate $\bar{z}_A^*$ and $\bar{z}_L^*$.

To begin, we obtained the maximum daily temperature data in 2000 ($T_{\text{max},2000}$) and used its floor value (i.e., the greatest integer that is less than or equal to $T_{\text{max},2000}$) as the initial values for $\bar{z}_A^*$ and $\bar{z}_L^*$.

Next, we ran the eco-evolutionary model (Model 3) for a period of five years using daily temperature data in 2000. By analyzing the output value of daily evolving $\bar{z}_A$ through linear regression, we can estimate the changing trend of $\bar{z}_A$ and $\bar{z}_L$. If the trend value is positive, it means that the value of $\bar{z}_A^*$ is too low and needs to be increased. Conversely, a negative trend value indicates that $\bar{z}_A^*$ is too high and needs to be decreased. To determine the optimal value of $\bar{z}_A^*$, we continually adjusted its value by 0.5 °C until the sign of the trend changed.

Once we determine the locally adapted optimal temperature for aphids ($\bar{z}_A^*$), we repeated the above procedures to estimate the locally adapted optimal temperature for ladybirds ($\bar{z}_L^*$) and verify if $\bar{z}_A^*$ remains the same. Based on $\bar{z}_A^*$ and $\bar{z}_L^*$, we calculated the other thermal performance parameters and obtained the thermal performance traits that best match the local temperature data in 2000 for each species. The estimated values of $\bar{z}_A^*$ and $\bar{z}_L^*$ for each geographic location are shown in Table 1 (main text).

References

Ge, X., Griswold, C. K. and Newman, J. A. (2023) Warmer and more seasonal climates reduce the effect of top-down population control: An example with aphids and ladybirds. *Functional Ecology*, **37**, 1604–1619.

Houle, D., Morikawa, B. and Lynch, M. (1996) Comparing mutational variabilities. *Genetics*, **143**, 1467–1483.

Islam, Y., Güncan, A., Zhou, X., Naeem, A. and Shah, F. M. (2022) Effect of temperature on the life cycle of *Harmonia axyridis* (Pallas), and its predation rate on the *Spodoptera litura* (Fabricius) eggs. *Scientific Reports*, **12**, 1–14.

Latimer, C. A., McGuigan, K., Wilson, R. S., Blows, M. W. and Chenoweth, S. F. (2014) The contribution of spontaneous mutations to thermal sensitivity curve variation in drosophila serrata. *Evolution*, **68**, 1824–1837.

Lynch, M. and Walsh, B. (1998) *Genetics and analysis of quantitative traits*. Sinauer Sunderland, 1 edn.

Satar, S., Kersting, U. and Uygun, N. (2005) Effect of temperature on development and fecundity of *Aphis gossypii* Glover (Homoptera: Aphididae) on cucumber. *Journal of Pest Science*, **78**, 133–137.
